# Altered polymerase theta expression promotes chromosomal instability in salivary adenoid cystic carcinoma

**DOI:** 10.1111/jcmm.17429

**Published:** 2022-06-21

**Authors:** Han Bai, Shilin Xia, Lei Zhu, Yan Dong, Chao Liu, Nan Li, Han Liu, Jing Xiao

**Affiliations:** ^1^ College of Stomatology Dalian Medical University Dalian China; ^2^ Clinical Laboratory of Integrative Medicine The First Affiliated Hospital of Dalian Medical University Dalian China; ^3^ Liaoning Province Key Laboratory of Organism Microecology and Disease Control Dalian China

**Keywords:** CEBPB, chromosomal instability, DNA repair, POLQ, SACC

## Abstract

Genomic instability (GIN) plays a key role in cancer progression. The disorders of polymerase theta (POLQ) were reported to contribute to GIN and progression in many cancers. Here, we found that POLQ over‐expression was related to salivary adenoid cystic carcinoma (SACC) progression and poor prognosis. Then, we investigated the role and mechanism of POLQ in the GIN in SACC. GIN was assessed by chromosome staining with DAPI and Giemsa, as well as qRT‐PCR of the mitosis‐related gene expression. Meanwhile, PCR‐SSCP was used to evaluate microsatellite instability. Modulation of POLQ expression increased chromosomal instability and enhanced the sensitivity to etoposide without impacting microsatellite stability. Mechanistically, POLQ regulated genome stability by promoting the expression of the error‐prone alt‐NHEJ‐related protein PARP1, and down‐regulating c‐NHEJ‐ and HR‐related proteins KU70 and RAD51. In vitro CCK, Transwell assays and in vivo murine xenograft models indicated that the PARP inhibitor olaparib suppressed SACC growth in the case of etoposide‐induced DNA damage. Bioinformatic analysis identified CEBPB as a potential POLQ‐regulating transcription factor. In summary, our research provides new insights into the mechanisms of SACC chromosomal instability and identifies new potential targets for SACC treatment.

## 1 INTRODUCTION

Salivary adenoid cystic carcinoma (SACC) is a slow growing, solid and malignant tumour that accounts for 1% of all head and neck tumours.^1^ SACC frequently shows local recurrence, adjacently perineural invasion and distantly hematogenous metastasis to lung, bone and liver.^2^ SACC is classified into three subtypes: cribriform, tubular and solid subtypes. The solid SACC shows a high propensity for relapse and poor prognosis compared with cribriform and tubular SACC.^3^ The gold‐standard treatment for primary SACC is radically surgical resection and postoperative radiotherapy. However, the patients treated with these strategies still suffer from local recurrence and distant metastasis, with the poor long‐term prognosis,^4^ because of the SACC progression which is the main reason of the poor efficacy of treatment.^5^ Therefore, the further understanding of the mechanisms of SACC progression is critical to identify the novel treatment strategies for controlling SACC progression and ultimate improving prognosis.

Tumours are characterized by genomic instability (GIN), which leads to a higher probability for genomic changes and the acquiring of deleterious mutations that may lead to tumour progression.^6^ GIN includes chromosomal instability (CIN) and microsatellite instability (MSI).^7^ In response to radiotherapy, most tumour cells exhibit an excessive GIN that results in cell apoptosis, but a small portion of tumour cells acquire non‐fatal GIN, which enhances tumour heterogeneity, evolution and progression.^8^ Recent studies identified the more genomic mutations in recurrent/metastatic adenoid cystic carcinoma (ACC) compared with primary ACC.^9^ Furthermore, more than half of SACC cases characterized with GIN had worse outcomes compared with the SACC cases without GIN.^10^ These studies indicate that GIN promotes SACC progression, thus, suggesting that the mechanism underlying GIN is key to inhibit SACC progression.

In normally proliferating cells, DNA damage or mitotic defects are repaired by multiple DNA repair pathways. In human cells, the double‐strain break (DSB) repair includes the major DNA repair manner, including the classical non‐homologous end joining (c‐NHEJ) and homologous recombination (HR) pathways, as well as the error‐prone repair manner, the alternative non‐homologous end‐joining (alt‐NHEJ) pathway. In mammals, the c‐NHEJ and HR pathways are the most precise DSB repair pathways that maintain chromosomal stability throughout the cell cycle. When c‐NHEJ and/or HR function are deficient, the alt‐NHEJ pathway will be activated.^11^ However, the alt‐NHEJ pathway is easy to cause GIN, such as DNA sequence loss, chromosome translocation and other events; so, it is also regarded as a main driver of cancer progression.^12^, ^13^

Polymerase theta (POLQ or POLθ) is an A‐family DNA polymerases that is widely expressed in eukaryotes and is highly expressed in tumour cells and some normal human tissues. POLQ functions as a key factor in the alt‐NHEJ pathway and participates in many cellular processes, including DNA repair and DNA replication.^14^ Increased expression of POLQ has also been detected in many kinds of tumours, such as lung cancer, colorectal cancer and gastric cancer.^15^ A higher expression of POLQ was significantly related to the poor survival in colorectal cancer and breast cancer .^16^, ^17^ Studies showed that POLQ‐addicted cells and cancers had a similar GIN feature, showing an increased prevalence of alt‐NHEJ‐related microhomology‐flanked deletions.^18^ POLQ depletion also reduces aberrant replication products and chromosomal rearrangements.^19^ However, there were few studies concerning the effect of POLQ on genomic stability, progression and prognosis in SACC. Here, we found that POLQ expression was related to SACC progression and poor prognosis. Through examining the function and regulatory mechanism of POLQ in SACC, we found that inhibiting POLQ or POLQ‐related pathways increased the sensitivity of SACC cells to DNA damage. Furthermore, we identified a new regulator of POLQ, the bZIP transcription factor CEBPB, which could bind to POLQ promoter. This study provides the evidence for inhibiting SACC progression and optimizing current therapies for the treatment of SACC.

## 2 MATERIALS AND METHODS

### 2.1 Patients and follow‐up

The data of the normal salivary glands and the SACC tissues that were surgically resected at Dalian Medical University (Dalian, China) between January 2001 and December 2013 were summarized and re‐examined according to our previous study.^20^ The pathological diagnosis in this study was conducted by two head and neck cancer pathologists (J. Xiao and L. Zhu). The written consents were obtained from patients or their guardians. This study was approved by the Dalian Medical University Ethics Committee (Dalian, Liaoning, China, ethics approval number: PJ‐KS‐KY‐2021‐271). The demographic and clinicopathological information of SACC patients are shown in Table 1.

**TABLE 1:** Correlation between patient clinicopathological data and POLQ expression in salivary gland tumours

Factor	No.	Average POLQ Index	POLQ IHC expression		
			Low	Increased	High
SACCs^**^	50	0.88	35 (70.0%)	9 (18.0%)	6 (12.0%)
Cribriform	15	0.07	14 (93.3%)	1 (6.7%)	0 (0%)
Tubular	17	0.35	14 (82.4%)	2 (11.8%)	1 (5.9%)
Solid^***^	18	2.06	7 (35.0%)	6 (30.0%)	5 (25.0%)
NSGs	20	1.00	20 (100%)	0	0
Age					
<60	28	0.79	22	3	3
≥60	22	1	13	6	3
Gender					
Male	22	0.77	14 (66.7%)	6 (28.6%)	2 (9.5%)
Female	28	0.96	21 (72.4%)	3 (10.3%)	4 (13.8%)
Tumour site					
Major SGs	31	0.35	24 (82.8%)	6 (20.7%)	1 (3.4%)
Minor SGs	19	1.74	11 (52.4%)	3 (14.3%)	5 (23.8%)

^**^ *p* < 0.01.

^***^ *p* < 0.001 (chi‐square test).

### 2.2 Cell culture

The human SACC cell lines, SACC‐83 and SACC‐LM, were generously gifted by Dr. Shenglin Li (Department of Oral and Maxillofacial Surgery, Peking University School and Hospital of Stomatology). SACC cell lines were cultured in RPMI 1640 (Gibco, C11875500BT). The 293 T cell line (FuHeng Biology, FH0244) was cultivated in DMEM basic (1×) medium (Gibco, C11995500BT). Both cell cultures were supplemented with 10% foetal bovine serum (Gibco, 10099‐141) and 100 U/ml penicillin/streptomycin (Gibco, 15140122).

### 2.3 Drug treatments

Salivary adenoid cystic carcinoma cells were treated with 500 μg/ml hygromycin B (Solarbio, H8080) to select POLQ‐OE positive cells. 3 μM etoposide (Sigma‐Aldrich, E1383) or dimethylsulfoxide (vehicle control) was used to treat cells for 8 h, and 58 μM olaparib (MedChemExpress, HY‐10162) or dimethylsulfoxide was used to treat cells for 48 h. Both etoposide and olaparib were dissolved according to the manufacturers' protocol.

### 2.4 Gene knockdown and over‐expression

POLQ knockdown in vitro and in vivo was achieved by PGPU6/GFP/Neo plasmids expressing shRNAs specifically targeting POLQ (Shanghai GenePharma Co., Ltd). The target sequences of the shRNAs were as follows: #1, 5′‐GCAGGAGAATGCAA GCCTACA‐3′; #2, 5′‐GGGACTTCCTAAAGCAGTTCT‐3′; and #3, 5′‐GGATATAT TTCCTGTCCAAGA‐3′. The PGPU6/GFP/Neo vector was used as the negative control (shControl) with an shRNA sequence without homology to any human gene. The pcDNA 3.1(Hygro) myc‐hPolQ‐Flag vector for POLQ over‐expression was a gift from Agnel Sfeir (Addgene plasmid #73132; http://n2t.net/addgene:73132; RRID: Addgene_73132).^21^ Cells were transfected with the indicated constructs using Lipofectamine® 3000 Transfection Reagent (Invitrogen, L3000015), in accordance with the manufacturer's protocol. After 24 h, cells were selected with antibiotics for 48 h, and then, harvested for subsequent experiments.

CEBPB knockdown in vitro was achieved by siRNA specifically targeting CEBPB (Shanghai GenePharma Co., Ltd). The sequences of the siRNA‐CEBPB and the negative control are described as follows, respectively: 5′‐GUGUACAGAUGAA UGAUAATT‐3′ (sense) and 5′‐UUAUCAUUCAUCUGUACACTT‐3′ (antisense), 5′‐UUCUCCGAACGUGUCACGUTT‐3′ (sense) and 5′‐ACGUGACACGUUCGGA GAATT‐3′ (antisense). The CEBPB wild‐type sequence was cloned into pcDNA3.1‐RFp vector; both the CEBPB over‐expression plasmid and control plasmid were constructed by Shanghai GenePharma Co., Ltd.

### 2.5 RNA extraction and quantitative real‐time PCR (qRT‐PCR)

RNA extraction and qRT‐PCR were performed as previously described.^22^ Total RNA was extracted from SACC cells by RNAiso Plus (Takara, 9109). The PrimeScript™ RT reagent Kit with gDNA Eraser (Perfect Real Time; Takara, RR047A) was used to synthesize cDNA using a T100™ Thermo Cycler (Bio‐Rad, 621BR18494). Equal amounts of cDNA were amplified by TB Green® Premix Ex Taq™ II (Tli RNaseH Plus; Takara, RR820A) using the QuantStudio™ 6 Flex Real‐Time PCR System (Thermo Fisher Scientific, 4484642) and the specific primers for human genes (Takara). The qRT‐PCR results were obtained from three independent replicates. The relative changes in gene expression were summarized based on the 2^−ΔΔ^CT method. All of the primer sequences are listed in Table S1.

### 2.6 Western blot

Protein concentrations of cell lysates were determined using a BCA protein assay (KeyGen, KGP902). Equal amounts of protein were separated by 8%, 10%, or 15% SDS‐PAGE and transferred onto nitrocellulose filter membranes (Solarbio, YA1710) or polyvinylidene difluoride membranes (GE, 10600023) by electroblotting. The membranes were blocked and then, incubated with primary antibodies at 4°C overnight. The primary antibodies are listed in Table S2. Anti‐GAPDH antibody and anti‐VINCULIN antibody were used as loading controls. After washing, the membranes were incubated with secondary antibodies. The blots were washed, and signals were visualized with WesternBright™ ECL (Advansta, K‐12045‐D50). Bands were detected by a ChemiDoc™ Imaging System (Bio‐Rad, 733BR2511). The secondary antibodies were HRP‐conjugated Goat Anti‐Rabbit IgG(H + L; ABclonal, AS014) and HRP‐conjugated Goat Anti‐Mouse IgG(H + L; ABclonal, AS003).

### 2.7 Haematoxylin–eosin staining

The histopathology examination of SACC xenograft tumour tissues was performed with haematoxylin–eosin staining. Briefly, after deparaffinization and rehydration, slides were stained with haematoxylin (Maixin Biotech, CTS‐1097) and eosin (Solarbio, G1100). The slides were then observed using an Olympus BX43 microscope (Olympus Corporation).

### 2.8 Immunohistochemistry

Immunohistochemical staining of paraffin‐embedded SACC and subcutaneous tumours of nude mice was performed using the DAB (3,3‐diaminobenzidine) kit (Maixin Biotech, DAB‐0031) in accordance with the manufacturer's instructions. Briefly, slides were deparaffinized and rehydrated, and endogenous peroxidase activity was deactivated using 3% H_2_O_2_ in distilled water. Citrate buffer was used for antigen retrieval at 100°C. Slides were blocked with non‐immune active goat serum (Maixin Biotech, SP KIT‐B2) and then, incubated with the primary antibodies against POLQ (Invitrogen, PA5‐69577, dilution 1:50), γH2AX (Cell Signalling Technology, 9718, dilution 1:250), CASPASE‐3 (Cell Signalling Technology, 9664, dilution 1:200) and Ki‐67 (Zenbio, 511390, dilution 1:400). Information on the primary antibodies is listed in Table S1. For negative controls, the primary antibody was excluded. The sections were then processed using the VECTORSTAIN ABC kit (Vector Labs, PK‐4001). The specimens were counterstained with haematoxylin (Maixin Biotech, CTS‐1097) and then, observed using an Olympus BX43 microscope (Olympus Corporation). Quantitative analysis of immunostaining was conducted as described previously.^22^

### 2.9 Xenograft mouse model

Male BALB/c nude mice (age 28–36 days) were obtained from the Vital River Laboratories Animal Technology Co., Ltd. All animal procedures were performed in accordance with the Guide for the Care and Use of Laboratory Animals and were approved by the Ethics Committee of Dalian Medical University (No. AEE20030). SACC‐83 and SACC‐LM cells (2 × 10^6^ cells) were implanted subcutaneously in the nude mice. Treatment started when the tumour size reached ~50 mm^3^.Mice bearing SACC‐83 tumours were randomly divided into two groups: the POLQ‐OE group (POLQ‐OE plasmid, 10 μg/50 mm^3^ injected into tumours every 3 days, five times) and the control group (the same volume of saline, injected into tumours every 3 days). Mice bearing SACC‐LM tumours were randomized into five groups for treatment as follows: olaparib group (olaparib, 50 mg/kg, i.p., q.d., 14 times); etoposide + olaparib group (etoposide, 10 mg/kg, i.p., three consecutive days every 11 days, six times + olaparib, 50 mg/kg, i.p., q.d., 14 times); etoposide group (etoposide, 10 mg/kg, ip, three consecutive days every 11 days, six times); shPOLQ + etoposide group (shPOLQ plasmid, 10 μg/50 mm^3^, injected into tumours every 3 days, five times) and control group (saline with the same volume by the same injection way on that day).

Tumour size was limited to less than 1000 mm^3^ in compliance with animal welfare regulations. After 14 days of treatment, the mice were euthanized with CO_2_, and lack of respiration and heartbeat were used as an indicator of death. The xenografts were measured and retrieved. The specimens were divided into two sets: a half was kept frozen and stored at −80°C, and the remaining half was prepared as a paraffin block.

### 2.10 Cell proliferation, cell migration and invasion assays

The Cell Counting Kit (CCK, TransGen Biotech, FC101) was used to detect cell proliferation in accordance with the manufacturer's protocol. Cells (1 × 10^4^) were seeded with 100 μl of different treatment medium per well in 96‐well plates. CCK was added to each well and incubated at 37°C for 2 h. The absorbance was measured at 450 nm using the SpectraMax®Plus384 Absorbance Microplate Reader (Molecular Device). The migration and invasion capacities of SACC cells were examined by Transwell chamber assays. Matrigel (BD Biosciences, 354480) was used for the invasion assay. Briefly, SACC cells (1 × 10^5^ cells) were suspended in serum‐free medium and seeded into the top chamber. The bottom chamber contained complete growth media. Cells were incubated for 48 h. Migrating or invading cells were stained with 0.4% crystal violet in methanol. The cells were photographed using an Olympus BX43 microscope (Olympus Corporation) and counted.

### 2.11 Immunofluorescence staining

Cover slips were treated with poly‐D‐lysine overnight before SACC cells were plated. After different treatments, cells were fixed with 4% paraformaldehyde in PBS for 20 min. Then, cells were permeabilized and blocked with goat serum with 0.3% Triton X‐100 at room temperature for 1 h. Then, samples were incubated with primary antibody (rabbit anti‐γH2AX, Abcam, 1:200) at 4°C overnight followed by one wash and then, incubation with secondary antibody (Alexa Fluor 488‐conjugated Affinipure Goat Anti‐Rabbit IgG(H + L), 1:200, Proteintech, SA00006‐2) for 1 h at room temperature. Cells were stained with DAPI (Solarbio, C0065) for 5–10 min, washed with PBS and then, examined using an Olympus IX71 microscope (Olympus Corporation). For nuclear fluorescence staining, the fixed cells were directly stained by DAPI before imaging.

### 2.12 Cytotoxicity assay

The MTT assay was performed to evaluate drug cytotoxicity in vitro. SACC‐83 cells were trypsinized and resuspended in fresh media in 96‐well plates. Cells were cultured overnight at 37°C and then, treated with various concentrations of etoposide (0, 0.01, 0.1, 1, 10, 100 and 1000 μM) and olaparib (0, 0.01, 0.1, 1, 10, 100 and 1000 μM) for 48 h. Cell viability was determined using the Cell Counting Kit (CCK, TransGen Biotech, FC101) in accordance with the manufacturer's protocol. Absorbance of each well at 450 nm was performed using the SpectraMax®Plus384 Absorbance Microplate Reader (Molecular Device). The Bliss method was employed to calculate IC_50_.

### 2.13 Bioinformatics analysis

The bioinformatics websites PROMO (http://alggen.lsi.upc.es/), Cistrome (http://cistrome.org/db/#/), JASPAR (http://jaspar.genereg.net/) and GEPIA (http://gepia.cancer‐pku.cn/) were used to predict potential binding sites in POLQ gene.

### 2.14 Dual‐luciferase reporter assay

The wild‐type POLQ promoter sequence was cloned into the GPL4‐Basic vector and the CEBPB wild‐type sequence was cloned into the pcDNA3.1‐RFP vector (Shanghai GenePharma Co., Ltd). For transfection, 293 T cells were cultured in 96‐well clear‐bottom plates until they achieved approximately 70% confluence. Cells were co‐transfected with the luciferase reporter vector, the pRL‐TK vector (Genepharma Corporation), either CEBPB over‐expression plasmid or control plasmid, and either the POLQ‐promoter over‐expression plasmid or control plasmid. After 48 h, firefly and Renilla luminescence were measured using the Dual‐Glo® Luciferase Assay System (Promega, E2920) in accordance with the manufacturer's instructions. The relative activity of Renilla luciferase was normalized to firefly luciferase activity.

### 2.15 Chromosomal aberration analysis

Chromosomal aberration analysis was performed on each group of SACC‐83 or SACC‐LM cells. In brief, cells were treated with 0.2 mg/ml colcemid (Biological Industrial, 12‐004‐1D) for 3 h. Next, 2 ml of PBS was slowly added to the medium, and the cell suspension was centrifuged at 90 *g* for 5 min. The supernatant was discarded and cells were incubated with KCl hypotonic solution of 75 mM at 37°C for 30 min. Cells were then fixed with a mixture of methanol and acetic acid (vol/vol = 3:1) and dropped onto microscope slides. Air‐dried cells were stained with Giemsa Stain Solution (Coolaber, SL7010) for 30 min and rinsed with deionized water. At least 30 metaphase spreads were scored in each group.

### 2.16 PCR‐single strand conformation polymorphism (PCR‐SSCP)

DNA extraction was performed using the TIANamp Genomic DNA Kit (Tiangen, DP304). The PCR reaction included 0.8 μg of DNA template, 1 μl of each primer of (10 μM), 5 μl of 10× Taq Buffer (Tiangen, ET101‐01‐01), 4 μl of dNTP Mixture (2.5 mM; Tiangen, CD117), 1 μl of Taq DNA Polymerase (2.5 U/μl; Tiangen, ET101‐01‐01) and double‐distilled water to 50 μl. Reaction mixtures were preheated at 94°C for 3 min, followed by 30 cycles of denaturation at 94°C for 30 s, annealing at 55°C for 30 s, extension at 72°C for 1 min and a final extension at 72°C for 5 min. The quality of PCR products was evaluated by agarose gel electrophoresis. The primer sequences are listed in Table S3.^23^

For SSCP, 5 μl of the PCR products mixed with 1 μl of 6× TriTrack DNA Loading Dye (Thermo Scientific™, SM1211) were loaded on a 1.0 mm 8% non‐denaturing PAGE gel and electrophoresed at room temperature, 80 V, for ~2 h. The gel was then, analysed by silver staining (Solarbio, G7210) and imaging. The shift of forhead bands compared with the control group after electrophoresis was considered as the positive MSI.

### 2.17 Statistical analysis

All data were statistically analysed with IBM SPSS statistics 20.0 (IBM Corp.). Data are shown as mean ± SEM. The data between two groups were compared by unpaired two‐tailed Student's *t*‐test. Data among multiple groups were compared by one‐way analysis of variance followed by Tukey's post hoc test. The relationship between POLQ expression and the clinicopathological features of SACC patients was determined by chi‐square test. The prognosis of SACC patients was analysed by Kaplan–Meier analysis. Microsoft Excel 2016 and GraphPad Prism 8.0 (GraphPad Software Inc.) were used for data and statistical analyses. All experiments were performed at least in triplicate. *p*‐value <0.05 was considered to indicate statistical significance.

## 3 RESULTS

### 3.1 POLQ was highly expressed in relatively malignant SACC cell lines and tumours

To examine the expression of POLQ in SACC, we first compared the median expression of POLQ in tumour and normal samples from the GEPIA website (http://gepia.cancer‐pku.cn/detail.php?gene=POLQ). Since POLQ was widely expressed in various human organs and tissues (Figure S1A), we extracted the median expression values from Figure S1A and drew a heatmap and histogram for POLQ expression in glandular epithelial cancers and the corresponding normal tissues (Figure S1B‐C). POLQ expressions were relatively higher in glandular epithelial cancers, including rectum adenocarcinoma, lung adenocarcinoma, breast invasive carcinoma (BRCA), prostate adenocarcinoma, ovarian serous cystadenocarcinoma (OV), cervical squamous cell carcinoma and endocervical adenocarcinoma, thymoma (THYM) and pancreatic adenocarcinoma compared with the normal tissues. These results implicated that POLQ over‐expression promoted glandular epithelial cancer. However, there were no data about POLQ expression in SACC, another kind of glandular epithelial cancer, available from database or articles. Therefore, it is supposed to explore the role of POLQ in SACC.

**FIGURE 1:**
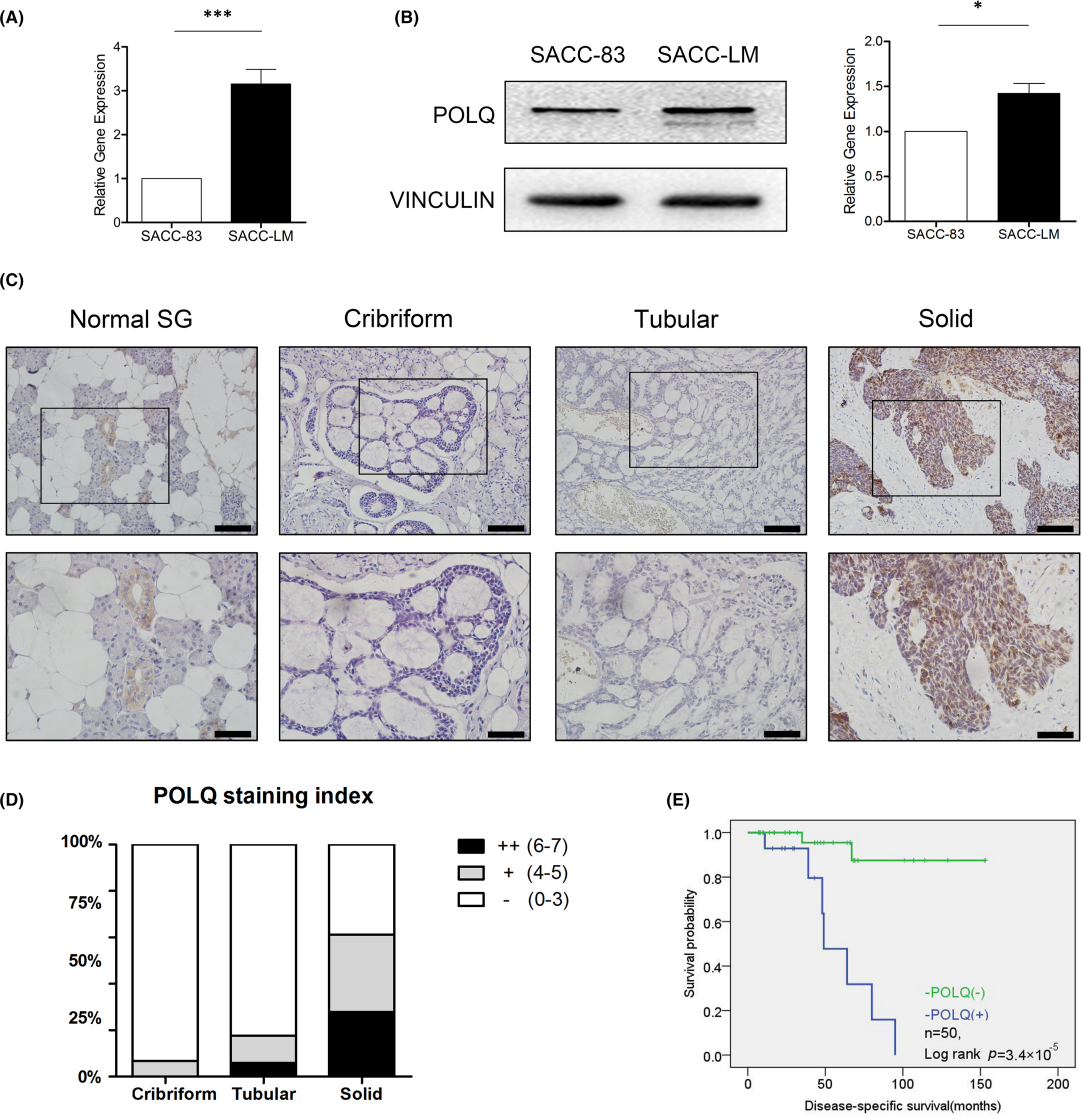
Expression of POLQ in SACC. (A) qRT‐PCR for POLQ mRNA in SACC‐83 and SACC‐LM cells. (B) Western blots and quantification of POLQ in SACC‐83 and SACC‐LM cells. (C) Immunohistochemistry analysis of POLQ in normal human salivary glands and the three subtypes (cribriform, tubular and solid) of SACC. Representative images are shown in two different magnifications. Scale bars, 100 and 50 μm. (D) Quantification of POLQ immunohistochemistry results in three types of human SACC. (E) Kaplan–Meier survival curve of 50 SACC patients with POLQ expression or without POLQ expression. Error bars on charts indicate SEM; *p < 0.05, ***p < 0.001, determined by unpaired, two‐tailed *t*‐test

We first investigated the expression levels of POLQ in a pair of SACC cell lines: the parental SACC‐83 cell line and the metastatic SACC‐LM cell line. The expression level of POLQ was higher in SACC‐LM cells than that in SACC‐83 cells (Figure 1A–B). Second, immunohistological analysis was performed for POLQ using human tissue arrays that included three types of SACC samples (15 of cribriform type, 17 of tubular type and 18 of solid type) and human normal salivary gland (NSG; 20 samples; Table 1; Figure 1C–D). In NSGs, we observed weakly cytoplasmic expression of POLQ in duct cells and undetectable expression of POLQ in abluminal (myoepithelial) cells and acini. In contrast, although both cribriform and tubular type SACC cases exhibited almost undetectable or low cytoplasmic expression of POLQ, most solid type SACC cases showed a higher level of cytoplasmic and nuclear POLQ staining (Figure 1C). The clinical information of tumour cases and results of POLQ immunohistochemistry staining was summarized in Figure 1D and Table 1. Low expression of POLQ (defined as POLQ staining index ≤3) was detected in 70% (35/50) of all SACCs and 100% (20/20) of NSGs. Low expression of POLQ was more frequently found in cribriform and tubular types (93.3%, 14/15; 82.4%, 14/17) compared with solid type (7/18, 35%). The frequency of the increased POLQ expression (defined as POLQ staining index 4–5) was 18% in SACCs (9/50), with 6.7% (1/15) in cribriform, 11.8% (2/17) in tubular and 30.0% (6/18) in solid types. The high level of POLQ expression (defined as POLQ staining index 6–7) was predominantly found in the poorly differentiated and high graded malignant SACCs (12%, 6/50), including the solid (25%, 5/18) and tubular types (5.9%, 1/17). While the high expression of POLQ was not found in NSGs.

Kaplan–Meier plots revealed that the patients with the higher POLQ expression had a worse survival probability than the patients with the lower POLQ expression: the survival probability rates were 56.3% and 94.1%, and the overall survival times were 36.9 and 55.4 months, respectively (*p* = 3.4 × 10^−5^; log‐rank test; Figure 1E). Collectively, these observations implicated an enhancement of POLQ on SACC progression.

### 3.2 Aberrant expression of POLQ promoted CIN in SACC

**FIGURE 2:**
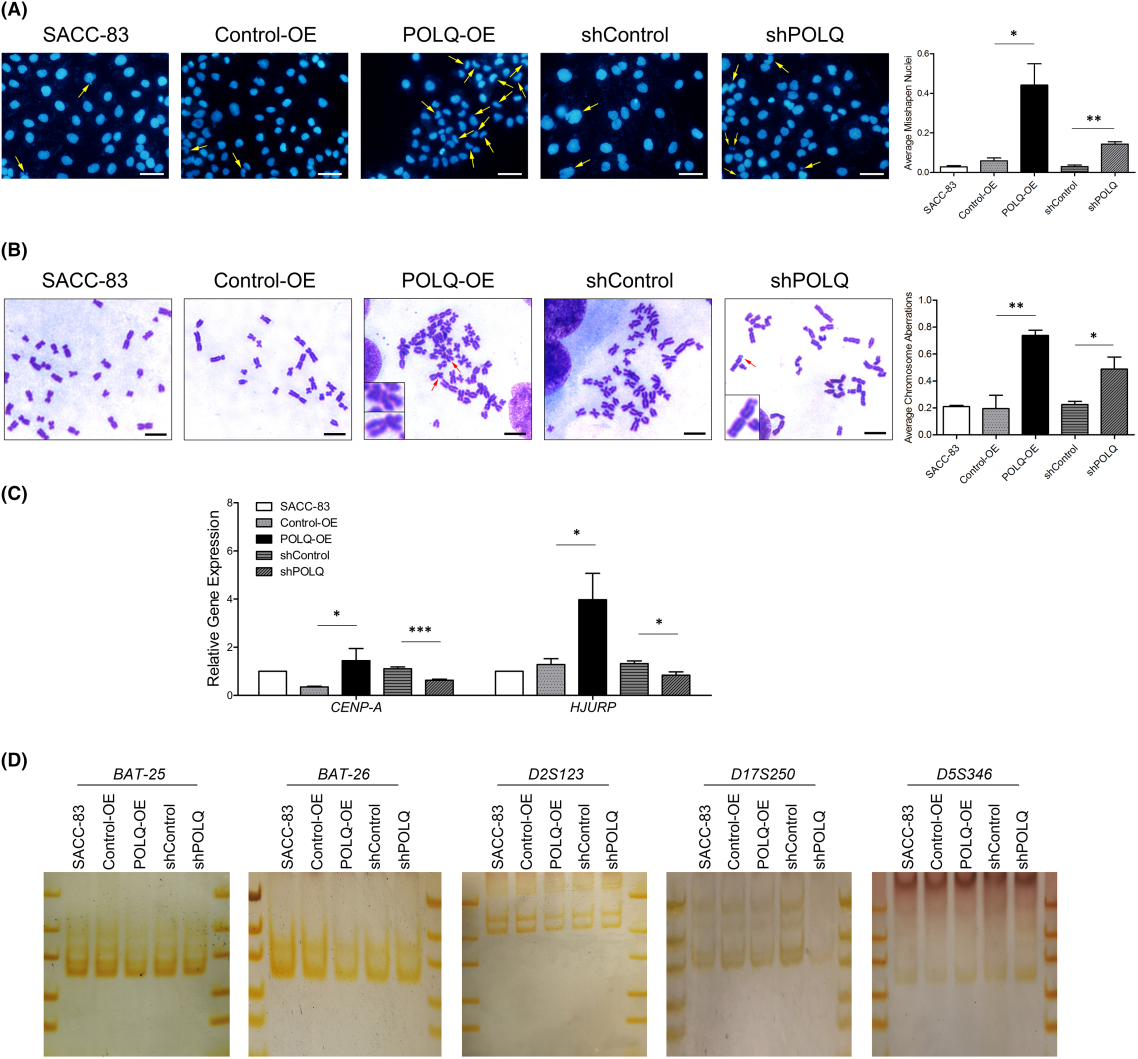
Effects of altered POLQ expression on GIN in SACC‐83 cells. (A) Representative images of cells with DAPI staining of nuclei (left panel) and quantification of nucleus deformation (indicated by yellow arrows in images) in the indicated cells (right panel). Scale bars, 50 μm. (B) Representative images of chromosomal aberrations (indicated by red arrows; left panel) and quantification of chromosomal aberrations in the indicated cells (right panel). Scale bars, 10 μm. (C) qRT‐PCR for CENP‐A and HJURP mRNAs in the indicated cells. (D) PCR‐SSCP analysis of *BAT‐25*, *BAT‐26*, *D2S123*, *D17S250* and *D5S346* in the indicated cells. Error bars on charts indicate SEM; *p < 0.05, **p < 0.01, determined by unpaired, two‐tailed *t*‐test

Then, we investigated whether POLQ played a role in genomic stability in SACC‐83, the lower lung metastatic line and SACC‐LM, the higher lung metastatic line. Because GIN included CIN and MSI, to determine whether POLQ influenced chromosomal stability, we first examined the occurrence of misshapen nuclei, chromosome structural aberrations and the expression of mitosis‐related genes in SACC cells with up‐regulated (POLQ‐OE) or suppressed (shPOLQ) POLQ expression (Figures 2A–C and S2). Altered POLQ expression noticeably induced nuclei deformations, such as micronuclei, crescents and abnormal sizes in SACC‐83 and SACC‐LM SACC cell lines (Figures 2A and S3A). POLQ over‐expression markedly increased the formation of misshapen nuclei, while POLQ deficiency slightly increased the proportion of misshapen nuclei. The frequency of chromosomal aberrations, including chromatid and chromosome breaks, dicentric structure, unpaired structure and chromosome radial structures, was increased in POLQ‐OE and shPOLQ SACC‐83 cells, as well as in shPOLQ SACC‐LM cells, in which the predominant lesions were chromatid and chromosome breaks (Figures 2B and S3B). These results suggested that the dysregulated expression of POLQ, both the over‐ and suppressed expression, promoted CIN.

To investigate whether POLQ plays a role in chromosomal stability through mitosis, we examined the mRNA levels of CENP‐A (a centromere marker) and the CENP‐A chaperone HJURP in SACC‐83 and SACC‐LM cell lines with POLQ over‐expression or POLQ down‐regulation. We found that POLQ‐OE SACC‐83 cells had the relatively high CENP‐A and HJURP mRNA levels compared with the control cells (Figure 2C). In contrast, the shPOLQ SACC‐83 cells displayed the relatively low CENP‐A and HJURP mRNA levels (Figures 2C and S3C).

Based on the effects of POLQ on chromosomal stability, to determine whether POLQ influenced microsatellite stability, we examined two mononucleotide markers, *BAT‐25* and *BAT‐26*, and three dinucleotide markers, *D2S123*, *D17S250* and *D5S346* (Figure 2D). These regions were amplified within parental, Control‐OE, POLQ‐OE, shControl and shPOLQ SACC‐83 cells with fluorescent multiplex PCR and their sizes were assessed by capillary electrophoresis. POLQ‐OE and shPOLQ SACC‐83 cells exhibited microsatellite stability at the five loci, suggesting that they were microsatellite stable cells (Figure 2D). These data suggested that POLQ influenced little microsatellite stability in SACC‐83 cells.

Together, these results suggested that the dysregulated POLQ expression increased the level of CIN, but not MSI, in SACC cell lines.

### 3.3 POLQ regulated SACC cell proliferation, migration and invasion in vitro and tumour size in vivo

**FIGURE 3:**
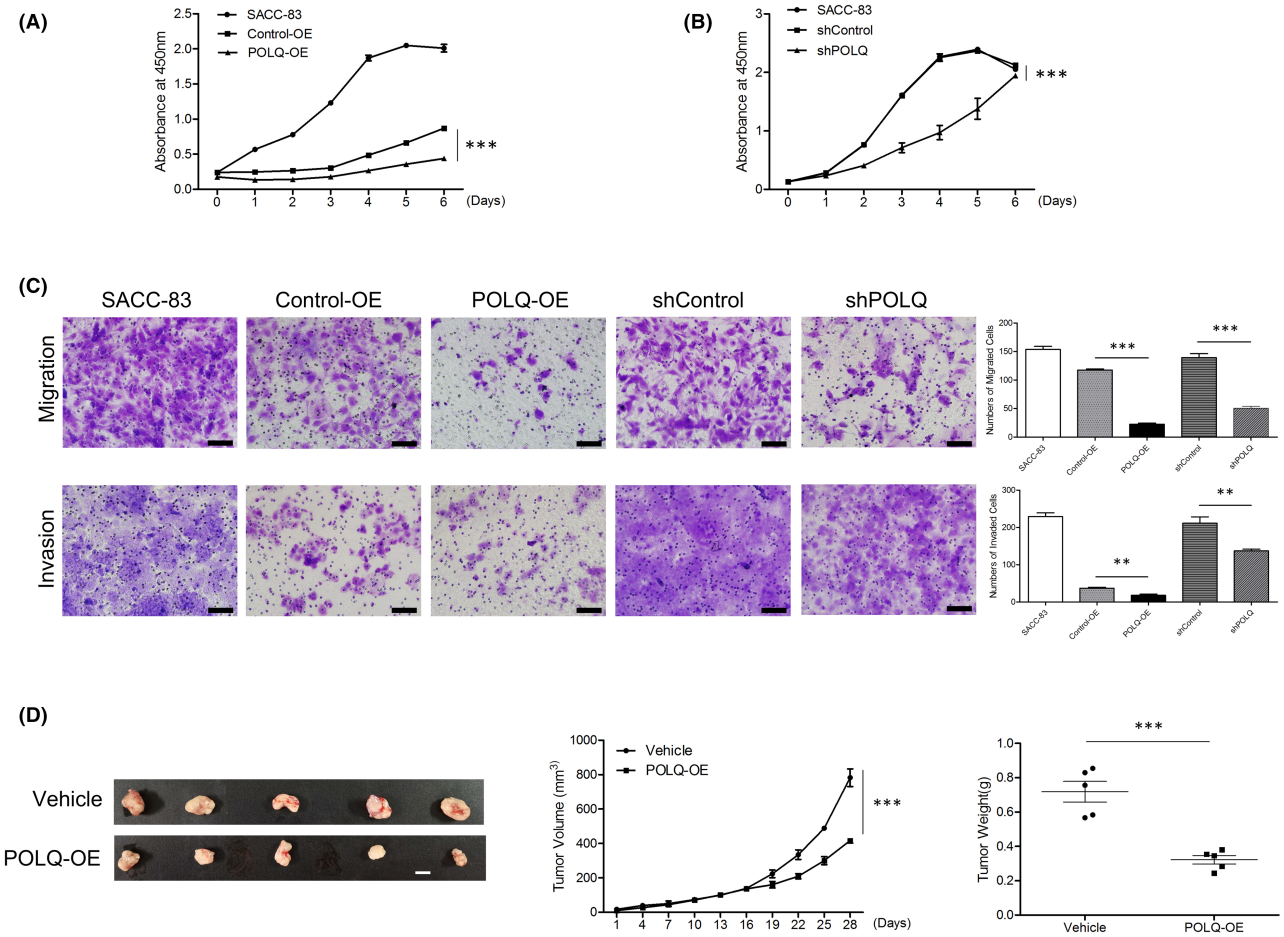
Effects of altered POLQ expression on cell proliferation, migration and invasion in vitro and tumour size in vivo. (A) Cell proliferation of the indicated cells was determined by CCK assay. (B) Cell proliferation of the indicated cells was determined by CCK assay. (C) Transwell migration (top panel) and invasion (bottom panel) assays with the indicated cells. Scale bars, 100 μm. (D) Effects of POLQ‐OE on SACC in vivo: tumour volume (left panel; scale bar, 1 cm), tumour growth (middle panel) and tumour weight (right panel). Error bars in graphs reflect SEM; **p < 0.01, ***p < 0.001, determined by unpaired, two‐tailed *t*‐test

To assess the role of POLQ in SACC malignancy, we examined proliferation, migration and invasion in POLQ over‐expressed and knock‐down SACC cell lines. POLQ‐OE and shPOLQ cells showed a suppressed proliferation compared with the corresponding control SACC‐83 and SACC‐LM cells (Figures 3A–B and S3D). Transwell migration and invasion assays revealed that POLQ over‐expression or deficiency in SACC‐83 or SACC‐LM cells decreased cell migration and invasion compared with the control SACC‐83 and SACC‐LM cells, respectively (Figures 3C and S3E).

To evaluate the effects of POLQ on SACC in vivo, we injected SACC‐83 cells with over‐expressed POLQ into nude mice. As shown in Figure 3D, the xenograft growth rate in the POLQ‐OE group was decreased compared with the control SACC‐83 xenografts. In addition, POLQ over‐expression dramatically decreased the size of SACC tumours. These results suggested that POLQ over‐expression inhibited SACC xenograft growth.

### 3.4 Altered POLQ expression increased the sensitivity of SACC cell lines to etoposide

**FIGURE 4:**
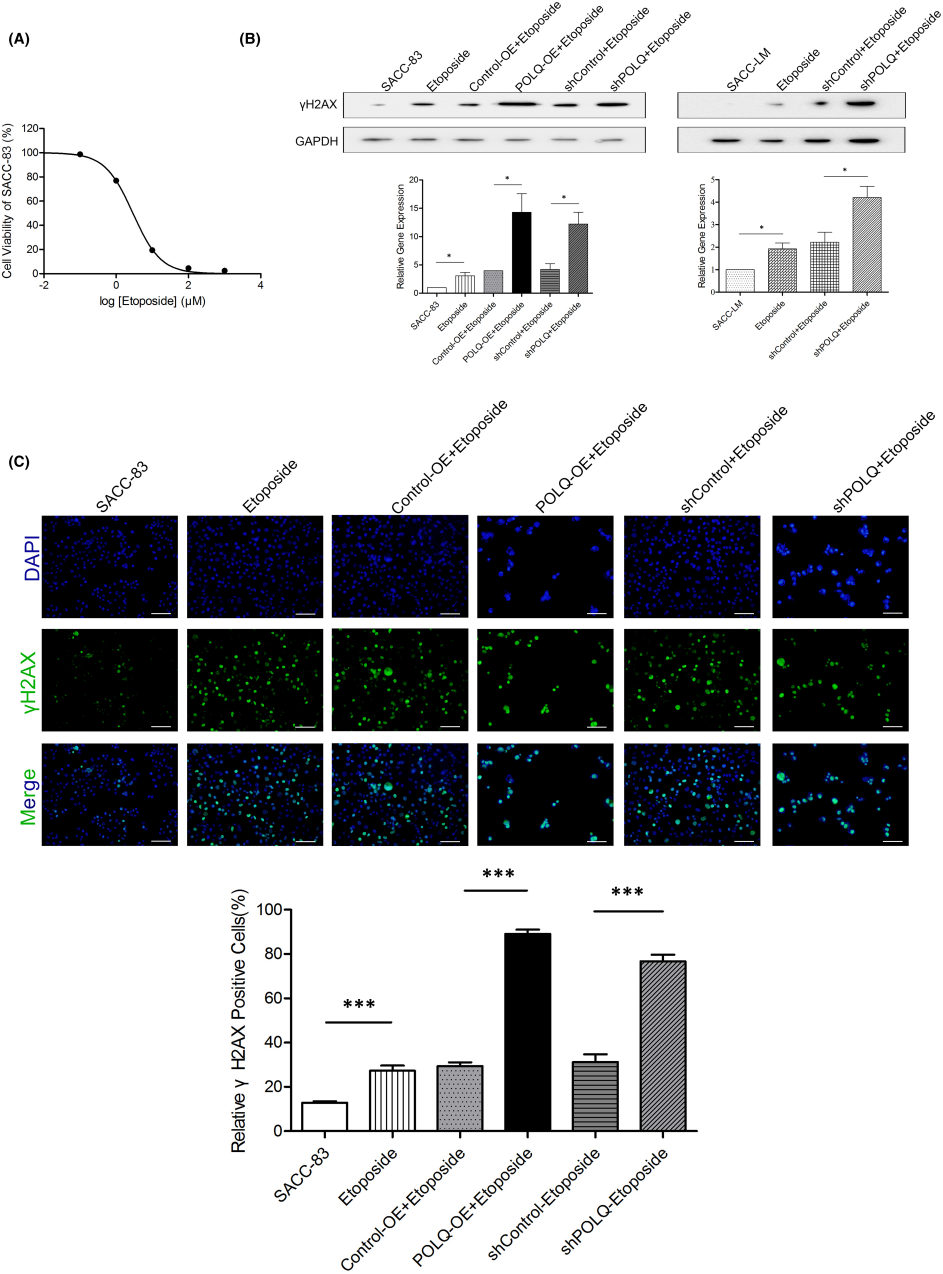
Effects of altered POLQ expression on DSBs under etoposide‐induced DNA damage. (A) Cell viability of SACC‐83 cells treated with etoposide from 0 to 1000 μM. (B) Western blots and quantifications of γH2AX in the indicated cells. (C) Immunofluorescence for γH2AX (top panel) in the indicated cells and quantification of positive cells (bottom panel). Scale bars, 100 μm. Error bars in graphs reflect SEM; *p < 0.05, ***p < 0.001, determined by unpaired, two‐tailed *t*‐test

Because POLQ was related to DNA repair, we evaluated the effect of altered POLQ expression in SACC cells under the DNA damaging agent, etoposide‐induced DNA damage. We first examined the effects of etoposide on the viability of SACC‐83 cells. Etoposide showed the high toxicity in SACC‐83 cells in a dose‐dependent manner, with an IC_50_ value of approximate 3 μM (Figure 4A). The levels of γH2AX, and the degrees of DSBs, were higher in POLQ‐OE and shPOLQ SACC‐83/LM cells treated with etoposide compared with those in control cells treated with etoposide (Figure 4B–C). These results implicated that the sensitivities of SACC‐83 and SACC‐LM cells to etoposide were increased when POLQ expression was disrupted.

### 3.5 Aberrant expression of POLQ enhanced the sensitivity of SACC to etoposide

**FIGURE 5:**
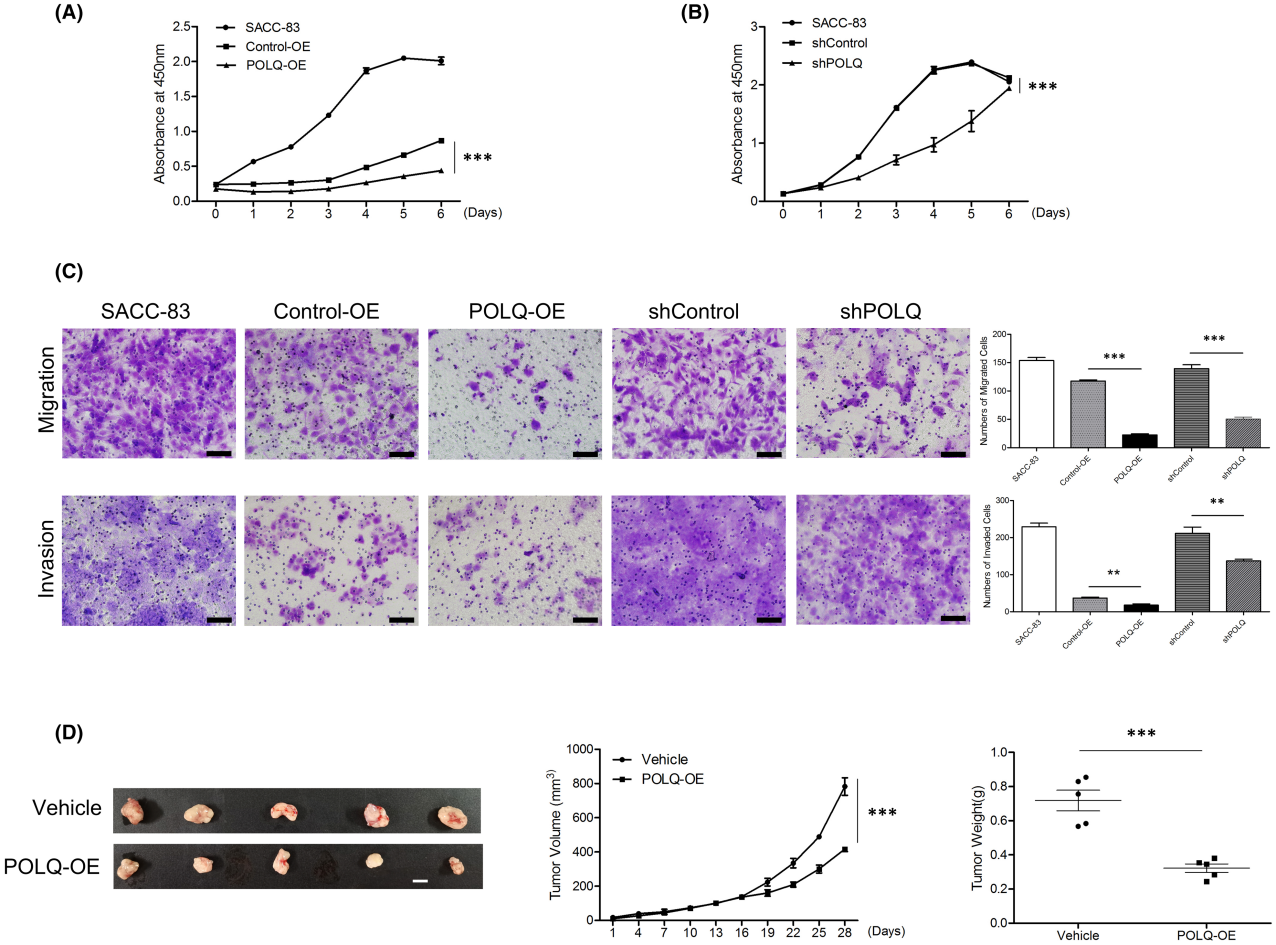
Effects of altered POLQ expression on chromosomal stability in vitro and tumour size in vivo under etoposide‐induced DNA damage. (A) Representative images of cells with DAPI staining of nuclei (left panel) and quantification of nucleus deformation (indicated by yellow arrows in images) in the indicated cells (right panel). Scale bars, 50 μm. (B) Representative images of chromosomal aberrations (indicated by red arrows; left panel) and quantification of chromosomal aberrations in the indicated cells (right panel). Scale bars, 10 μm. (C) Effects of shPOLQ on SACC under etoposide‐induced DNA damage conditions in vivo: tumour volume (left panel; scale bar, 1 cm), tumour growth curve (middle panel) and tumour weight (right panel). Error bars in graphs reflect SEM; *p < 0.05, **p < 0.01, ***p < 0.001, determined by unpaired, two‐tailed *t*‐test

Our results showed that the aberrant expression of POLQ increased DSBs in SACC cell lines under the condition of etoposide‐induced DNA damage. To evaluate the POLQ function and regulatory mechanism on CIN under DNA damage, we first detected the level of CIN in POLQ‐OE and shPOLQ SACC cell lines treated with etoposide of 3 μM for 8 h by counting the number of cells with aberrant nucleus shapes (Figures 5A and S4A). Aberrant expression of POLQ reamrkably induced the formation of deformed cell nuclei in SACC‐83 and SACC‐LM cells with etoposide treatment. More misshapen nuclei, mainly consisted of crescents and abnormal size, were observed in POLQ‐OE cells treated with etoposide compared those observed in etoposide‐treated control transfected cells. In contrast, more misshapen nuclei, predominantly consisted of micronuclei and abnormal size, were observed in the shPOLQ cells treated with etoposide compared with the corresponding etoposide‐treated controls.

We also analysed the frequency of chromosomal aberrations in cells with altered POLQ expression treated with etoposide (Figures 5B and S4B). The frequencies of chromosomal aberrations increased in POLQ‐OE and shPOLQ SACC‐83 cells, as well as in shPOLQ SACC‐LM cells following etoposide treatment compared with their corresponding controls.

POLQ over‐expression was related to poor prognosis in many cancers.^16^, ^17^ We, therefore, examined the potential efficacy of POLQ inhibition under DNA damage in SACC tumours. We established xenografts in athymic mice using shPOLQ SACC‐LM and control cell lines, and treated mice with etoposide to determine the impact of altered POLQ expression on SACC tumours under DNA damage. We found that POLQ suppression combined with etoposide treatment showed greater efficacy in reducing tumour growth compared with etoposide alone (Figure 5C).

### 3.6 POLQ negatively regulated RAD51 and KU70 expressions

Our results showed that both over‐expressed and suppressed POLQ induced CIN, even independent of etoposide treatment. However, why the over‐expression of POLQ was related to poor prognosis in cancer and the differential mechanisms between POLQ over‐expressing and suppressing cells were not clear. CIN is primarily associated with defects in the DNA damage response. Therefore, we evaluated the effects of POLQ on DNA damage repair pathways.

**FIGURE 6:**
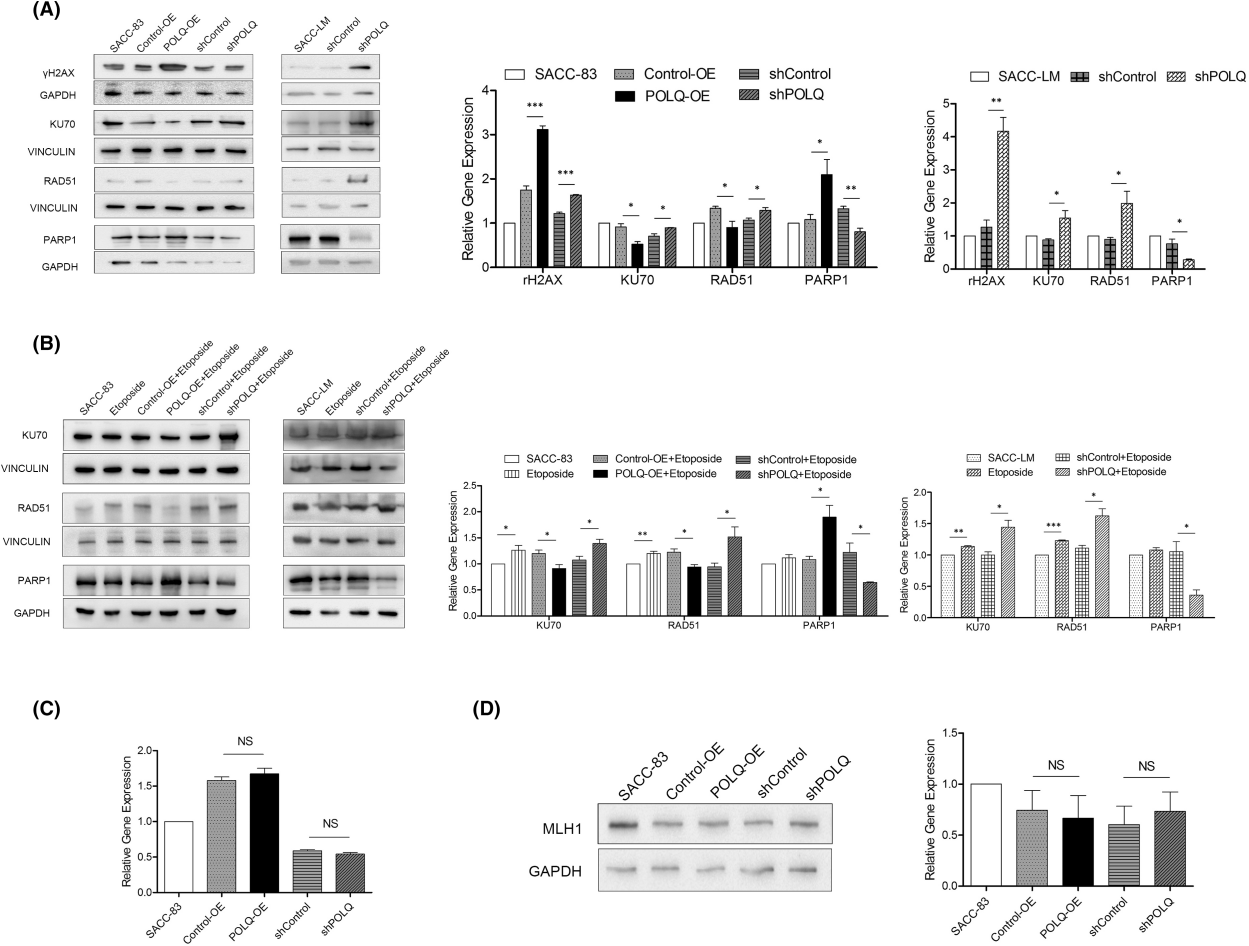
Effects of altered POLQ expression on DNA damage repair pathways. (A) Western blots and qualifications of γH2AX, RAD51, KU70 and PARP1 in the indicated cells. (B) Western blots and qualifications of γH2AX, RAD51, KU70 and PARP1 in the indicated cells under etoposide‐induced DNA damage. (C) qRT‐PCR for MLH1 mRNA and protein in the indicated cells. (D) Western blots and qualification of MLH1 protein in the indicated cells. Error bars in graphs reflect SEM. Error bars in graphs reflect SEM; *p < 0.05, **p < 0.01, ***p < 0.001, determined by unpaired, two‐tailed *t*‐test

In POLQ‐OE and shPOLQ SACC cells, γH2AX levels were increased compared with the corresponding control groups (Figure 6A). The levels of RAD51, a key protein of HR pathway, and KU70, a key protein of c‐NHEJ pathway, were decreased in SACC‐83 cells with over‐expressed POLQ, and increased in both the suppressed POLQ cells (Figure 6A). The levels of RAD51 and KU70 in shPOLQ SACC‐83/LM cells treated with etoposide were higher than those in corresponding control cells treated with etoposide alone and those in POLQ‐OE SACC‐83 cells were lower than corresponding control cells (Figure 6B). PARP1, the key protein of the alt‐NHEJ pathway, was up‐regulated in shPOLQ SACC‐83 and SACC‐LM cells, and down‐regulated in SACC‐83 cells with POLQ over‐expression in both etoposide treated and absent conditions (Figure 6A–B). POLQ affected MLH1 little, a key regulator in the mismatch repair pathway that regulated microsatellite stability, in SACC‐83 cells (Figure 6C‐D). These results implicated that although both POLQ over‐expression and suppression induced CIN, the mechanisms were differential. Since the expression level of RAD51, KU70 and PARP1 have been utilized to predict the activities of HR, c‐NHEJ and Alt‐NHEJ pathways, respectively,^24^, ^25^, ^26^, ^27^, ^28^ we speculated that POLQ negatively regulate HR and c‐NHEJ, but positively regulate alt‐NHEJ in SACC cell lines. In addition, etoposide treatment enhanced the effects of DNA damage by POLQ suppression on SACC.

### 3.7 The PARP1 inhibitor olaparib improved the sensitivity of SACC to etoposide

**FIGURE 7:**
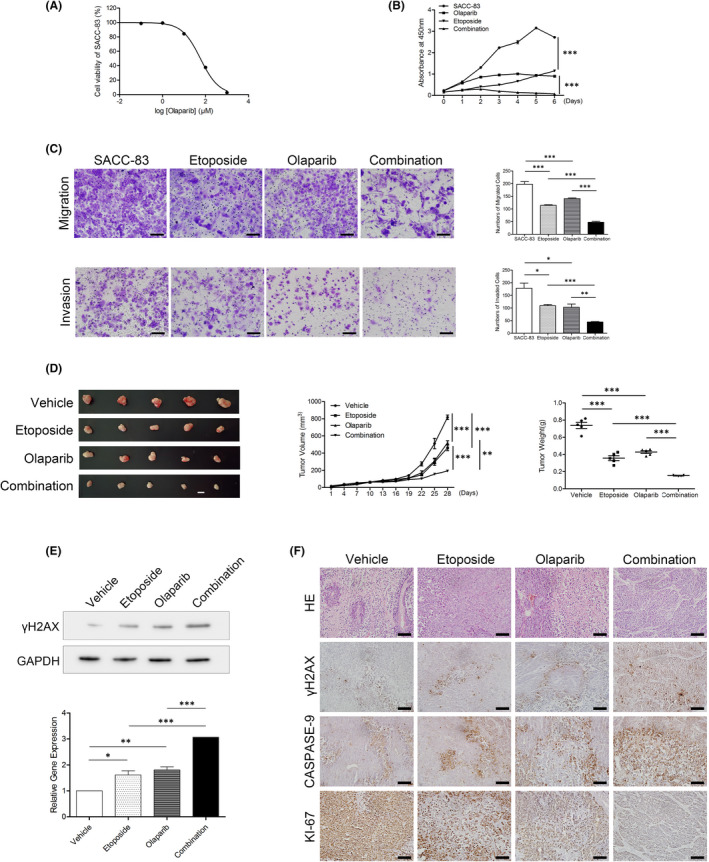
Effects of treatment with olaparib under etoposide‐induced DNA damage on SACC‐83 cells in vitro and xenograft tumours in vivo. (A) Cell viability of SACC‐83 cells treated with olaparib from 0 to 1000 μM. (B) Cell proliferation of the indicated cells was determined by CCK assay. (C) Transwell migration (top panel) and invasion assays (bottom panel) with the indicated cells. Scale bars, 100 μm. (D) Effects of olaparib on SACC under etoposide‐induced DNA damage in vivo: tumour volume (left panel; scale bar, 1 cm), tumour growth curve (middle panel) and quantitation of tumour weight (right panel). (E) Western blots and quantification of γH2AX in the xenografts treated with etoposide and olaparib alone, and in combination. (F) Haematoxylin–eosin staining and immunohistochemistry analysis of γH2AX, CASPASE‐3 and KI‐67 in SACC‐LM xenografts treated with etoposide and olaparib alone, and in combination. Scale bars, 100 μm. Error bars in graphs reflect SEM; *p < 0.05, **p < 0.01, ***p < 0.001, determined by unpaired, two‐tailed *t*‐test

As our results suggested a correlation between POLQ and PARP1, we explored the effect of a treatment targeting PARP1 on SACC tumorigenesis. We treated SACC‐83 cells with olaparib, a PARP1 inhibitor, to examine the viability of SACC‐83 cells. Olaparib was highly toxic to SACC‐83 cells in a dose‐dependent manner, with an IC_50_ value of approximate 58 μM (Figure 7A).

To evaluate the role of olaparib under DNA damage, we evaluated the proliferation, migration and invasion of SACC‐83 cell lines treated with etoposide and olaparib alone and in combination. Although Etoposide and olaparib alone or in combination could repress the proliferation, migration and invasion of SACC‐83 cells, the combined treatment had the most potentially inhibitory effect (Figure 7B–C).

To investigate the efficacy of olaparib under DNA damage in vivo, we injected SACC‐LM cells subcutaneously into nude mice to establish a SACC xenograft mouse model which were intraperitoneally injected etoposide and/or olaparib. As expected, the tumour growth in athymic mice with the treatment of etoposide and/or olaparib was greatly suppressed, among which the average volume of tumours with the combined treatment was the smallest (Figure 7D). Actually, the level of γH2AX in the combination treatment group was the highest among all treatment groups (Figure 7E–F). Although all treatments inhibited proliferation, as determined by Ki‐67 staining, and induced cell apoptosis, as shown by the percentage of CASPASE‐3 positive cells, the proliferation repression and apoptosis induction in the combined treatment group were more s pronounced than those in the single treatment groups (Figure 7F). Collectively, these data confirmed that SACC tumours with olaparib treatment were more sensitive to DNA damage in vitro and in vivo.

### 3.8 The CEBPB transcription factor bond to POLQ promoter in SACC

**FIGURE 8:**
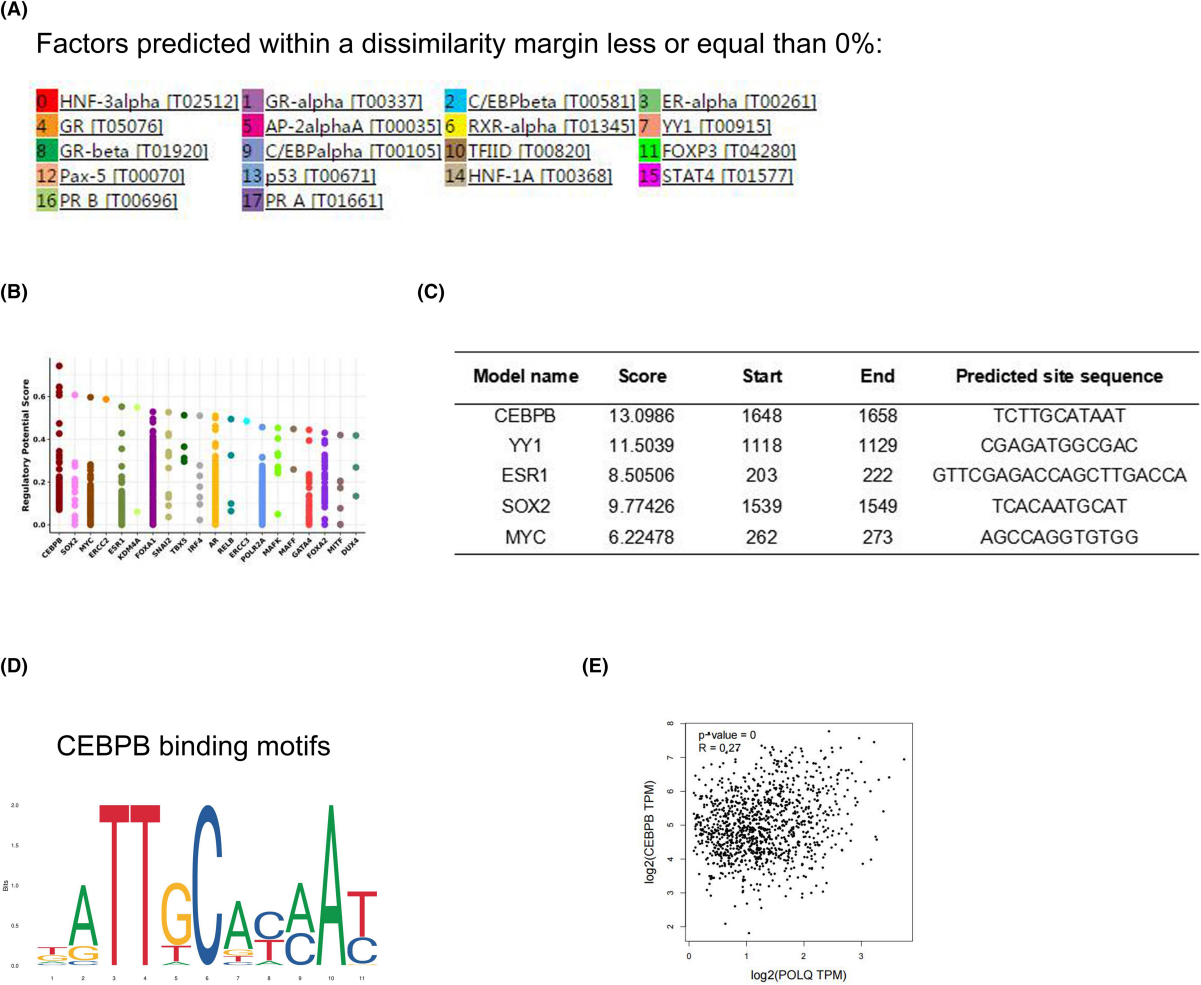
Analysis of transcription factors that regulated POLQ expression. (A) The PROMO prediction web tool identified potential transcription factors that regulated POLQ transcription. (B) The Cistrome Data Browser prediction web tool revealed the transcription factors with the high possibilities binding to the POLQ gene. (C) The JASPAR prediction web tool identified the transcription factors with the high scores for binding to POLQ promoter. (D) Sequence logo of the specific binding motifs of CEBPB. (E) Data in the GEPIA database showed a positive correlation between CEBPB and POLQ expression in BRCA. Colour images are available online

Our results showed that the up‐regulated POLQ positively was correlated with CIN and the poor prognosis of SACC. We elucidated the mechanism underlying POLQ over‐expression in SACC. Most polymerases are translated from protein‐encoded transcripts,^29^, ^30^ so, we speculated that transcription factors regulated POLQ expression by binding the promoter. Analyses using the PROMO prediction web‐based tool identified various transcription factors that were predicted to have a high affinity for POLQ promoter, such as CEBPbeta (also known as CEBPB), ER‐alpha (also known as ESR1) and YY1 (http://alggen.lsi.upc.es/cgi‐bin/promo_v3/promo/promoinit.cgi?dirDB=TF_8.3; Figure 8A). Cistrome Data Browser showed that CEBPB, SOX2 and MYC had the potentially highest scores for POLQ binding (http://dbtoolkit.cistrome.org/?specie=hg38&keyword=POLQ&factor=factor&distance=100k#plot_result; Figure 8B). JASPAR prediction showed that CEBPB also had a very high score for POLQ promoter binding (Figure 8C). Figure 8D showed the specific binding motifs in POLQ promoters (https://jaspar. genereg.net/matrix/MA0466.1/). We found that POLQ expression was positively correlated to that of CEBPB in BRCA (*p* < 0.05) in the GEPIA database (http://gepia.cancer‐pku.cn/detail.php?clicktag=correlation; Figure 8E).

### 3.9 The regulatory effects of CEBPB on POLQ in SACC cell lines

**FIGURE 9:**
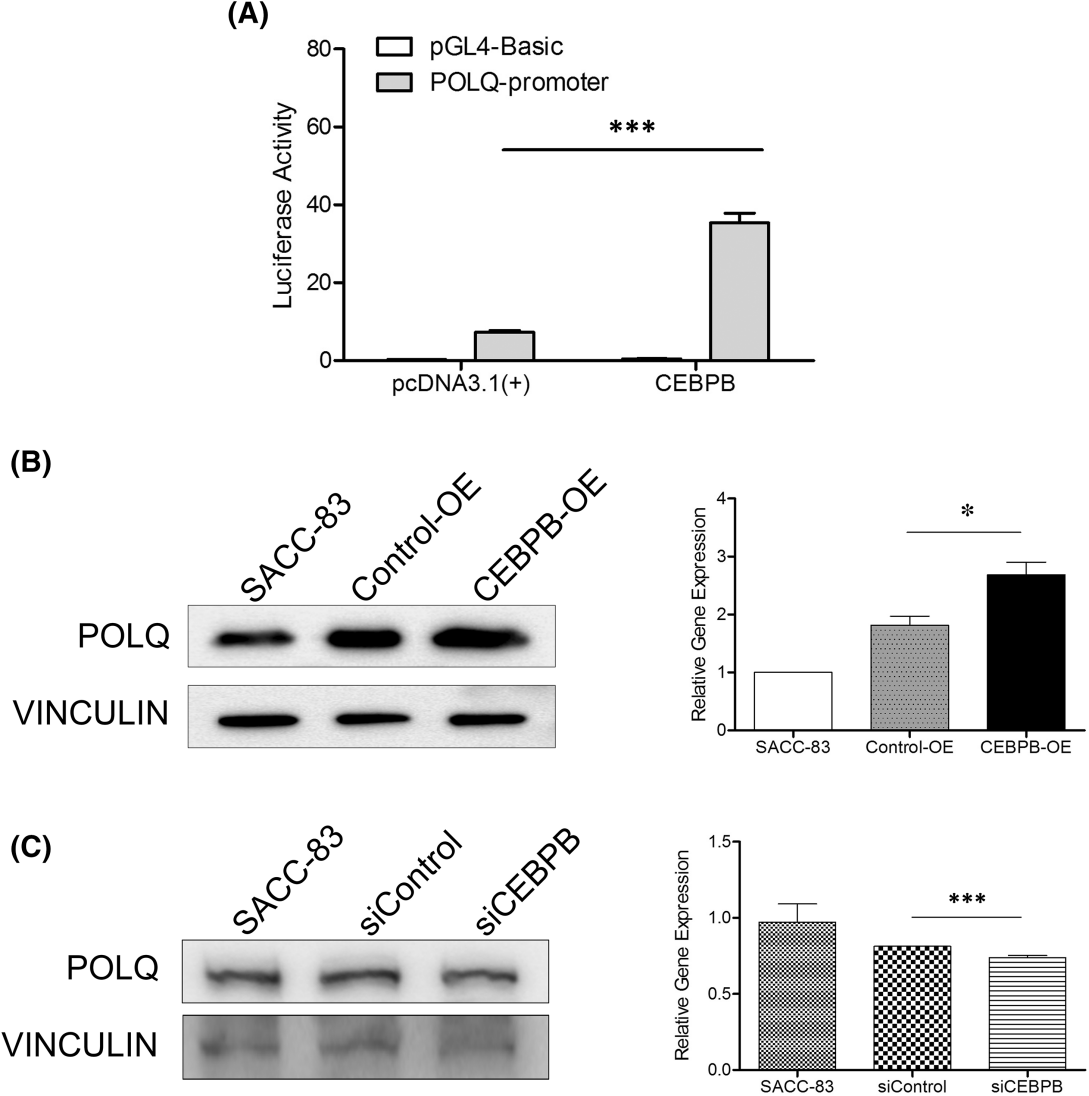
CEBPB regulated POLQ expression in SACC cells. (A) Dual‐luciferase reporter assay suggested that CEBPB positively regulated POLQ promoter activity. (B) Western blots and quantification of POLQ in the indicated SACC‐83 cells. (C) Western blots and quantification of POLQ in the indicated SACC‐83 cells. Error bars in graphs reflect SEM; *p < 0.05, ***p < 0.001, determined by unpaired, two‐tailed *t*‐test

To examine whether POLQ expression was regulated by CEBPB, we performed a dual‐luciferase reporter assay. The results showed that ectopic expression of CEBPB induced luciferase activity driven by the POLQ promoter (Figure 9A). To further determine the regulatory effect of CEBPB on POLQ in SACC, we performed in vitro experiments in SACC‐83 cells with the altered expression of CEBPB and POLQ. Western blots showed a slight increase or decrease in POLQ expression in response to the up‐ or down‐regulation of CEBPB (Figure 9B–C).

## 4 DISCUSSION

As a slow growing malignant tumour hard to be diagnosed,^31^, ^32^ SACC is considered as a biologically deceptive and frustrating head and neck tumour. SACC (especially the solid pattern type) displays heterogeneity with aggressive behaviour and treatment resistance, which lead to a poor long‐term prognosis.^33^ GIN is a characteristic of human cancers. The presence of GIN in most SACC cases and the higher GIN levels in recurrent/metastatic ACC^1^ suggest that GIN is an important driver in SACC progression. Therefore, we examined the GIN‐related POLQ expression in SACC. Bioinformatics analysis suggested that POLQ promote the progression of most glandular epithelial cancers, however, there was no data on SACC reported (Figure S1). POLQ expression in the highly metastatic SACC cell line SACC‐LM and solid SACC was higher than thoes in SACC‐83 cells (Figure 1A–B) and other types of SACC, respectively (Figure 1C–D). POLQ over‐expression was related to the poor SACC prognosis (Figure 1E). These findings suggest that POLQ promotes SACC progression.

Whether POLQ, the key factor in the alt‐NHEJ error‐prone repair pathway, promotes or inhibits GIN requires to be elucidated. Some studies showed that cells lacking POLQ exhibited the hypersensitivity to various DNA damaging agents,^34^ implicating that POLQ inhibited GIN. However, other evidences suggested that POLQ over‐expression increased DNA damage.^17^ A recent review showed that the alt‐NHEJ pathway mediated by POLQ had the characteristics of ‘error guaranteed’, and the aberrant POLQ expression could cause GIN: when POLQ was down‐regulated, DNA repair capability was decreased and GIN increased; in contrast, when POLQ was over‐expressed, the alt‐NHEJ pathway was over‐activated, and the error prone repair led to a large number of genomic scars and GIN increased.^24^ Thus far, few study concerned the relationship between POLQ and GIN in SACC. In this study, we examined the relationship between POLQ and GIN in SACC. DNA repair defects can lead to chromosome breaks, which induce nuclear dysmorphology, including the precursor structures of CIN, such as crescent, and the apoptosis‐related morphological changes, such as micronucleus and abnormal size.^35^, ^36^ We found a large number of apoptosis‐related morphological changes in SACC cells with the aberrant POLQ expression. We further found a higher ratio of misshapen nuclei in POLQ abnormally expressed cells, though the prolonged culture might reduce POLQ plasmid transfection efficiency. The more crescents, the fewer micronuclei and the nuclei with abnormal size, were observed in POLQ over‐expressed cells, while the more micronuclei and the nuclei with abnormal size were observed in POLQ‐deficient cells. We speculated that even if POLQ plasmid transient transfection efficiency faded, the non‐lethal CIN in ‘gene scars’ caused by the high POLQ expression mediated error‐prone alt‐NHEJ was still detectable. POLQ‐depleted cells were induced to apoptosis because of the limited DNA repair capability or continued to proliferate because of the recurrence of POLQ expression; so, CIN was just slightly increased. Because the misshapen nuclei are related to DNA damage and CIN, and as the physical properties of the nucleus depend largely on the chromosomes,^37^ we examined chromosome structures in POLQ abnormally expressed SACC cell models. Aberrant expression of POLQ increased chromosomal aberrations in SACC cells. These findings indicated that the excessive CIN resulted in the massive apoptosis in POLQ abnormally expressed SACC cell models.

We investigated the effect of POLQ on SACC mitosis by detecting CENP‐A and HJURP expression. Centromere protein A (CENP‐A) is a centromere specific variant of canonical histone H3 and indispensable for cancer progression.^38^ Over‐expression of CENP‐A cause CIN‐related mitosis defects and centromere dysfunction, which are the hallmarks of cancer.^38^ Holliday junction recognition protein (HJURP), the chaperone of CENP‐A, deposits CENP‐A at centromeres.^38^ HJURP is over‐expressed and related to the poor prognosis in some cancers.^38^ The loss of CENP‐A or HJURP leads to cell senescence,^39^, ^40^ and the depletion of CENP‐A causes mouse embryonic lethality.^41^ We found that POLQ positively regulated *CENP‐A* and *HJURP* transcription, suggesting that POLQ influenced chromosomal stability and SACC progression by regulating mitosis. These findings suggested that POLQ regulated chromosomal stability through impacting DNA damage. Furthermore, POLQ impact microsatellite stability little (Figure 2D). Mismatch repair defect is an important reason of MSI, and MLH1 expression in SACC was also impacted little by POLQ (Figure 6C). Previous studies have shown microsatellite stability in ACC.^9^ These results further prove that POLQ works as a regulator of CIN, instead of MSI in SACC.

We found that POLQ over‐expression inhibited SACC cell proliferation, migration and invasion at 48 h in vitro and SACC xenograft growth rates in vivo. The inhibited cell proliferation, migration and invasion by POLQ depletion indicated that POLQ was involved in the maintenance of chromosomal stability and cell survival. The 24 h cell migration and invasion ability were also inhibited by the aberrant expression of POLQ (data not shown), indicating that the migration and invasion were directly affected by POLQ in SACC.

At present, the non‐surgical treatment of SACC is radiotherapy. Radiotherapy is similar to topomerase II inhibitor etoposide–induced chromosomal instabilities, which are mainly repaired by the alt‐NHEJ pathway.^42^, ^43^ Thus, we used topomerase II inhibitor etoposide to simulate radiotherapy. Etoposide is used in the treatment of cancers because of its ability of inducing DNA damage.^44^ We found that etoposide suppressed cell proliferation in a concentration‐dependent manner in SACC‐83 cells. The function of POLQ in maintaining the chromosomal stability in SACC indicated the potential of POLQ as a therapeutic target for SACC. Previous studies indicated that POLQ inhibition increased cell sensitivity to many DNA damaging agents,^34^ while the stable POLQ over‐expression inhibited cell sensitivity to etoposide and hydroxyurea.^45^ We, therefore, examined the sensitivity of POLQ over‐expressing or inhibited cells to etoposide. As a high dose of etoposide may induce too many irreparable DSBs, we used approximately half of the maximally inhibitory concentration (IC_50_) to avoid inducing too many DNA damages to overwhelm POLQ function. We found that whether POLQ expression was increased or decreased, the level of CIN was increased in SACC cell lines under etoposide‐induced DNA damage in vitro. In addition, the combination of POLQ and etoposide inhibited SACC xenograft growth in vivo to a much greater extent than with etoposide alone. These results suggest the possibility of POLQ‐targeting therapy in SACC.

We also found both over‐expression and inhibition of POLQ induced DSBs in SACC, but the reason why both POLQ over‐expression and inhibition decrease SACC cell survival and increase DNA damage was unclear. Thus, we examined the different effects between POLQ over‐expression and inhibition on the expressions of key factors representing c‐NHEJ, HR and alt‐NHEJ pathways. The results showed that POLQ negatively regulated KU70 and RAD51, but positively regulated PARP1, implicating that POLQ elevated chromosomal stability by inhibiting c‐NHEJ and HR activity, while promoting error‐prone alt‐NHEJ activity. We will investigate the functional relevance of POLQ in DNA repair pathways in future studies.

Because of the low fidelity and high mutation, the alt‐NHEJ repair pathway is considered as the main driving force of CIN in human cancer.^12^, ^46^ Studies have shown that high expression of POLQ promotes CIN, such as genomic signal deletions and insertions and chromosome translocation, contributing to tumour progression.^12^, ^13^, ^15^ According to above results, we speculate that when POLQ is over‐expressed, alt‐NHEJ will be over‐activated. Due to the error prone characteristics of alt‐NHEJ, a large number of random CINs are formed, including the lethal CIN and non‐lethal CIN. POLQ over‐expressing cells with lethal CIN will undergo apoptosis immediately, and the remaining POLQ over‐expressing cells with non‐lethal CIN are survive from natural selection. These cells continue to proliferate and promote SACC progression. In contrast, when POLQ expression is decreased, DNA repair capability is decreased, so some DNA breaks can not be repaired and cells with the unrepaired DNA damage will undergo apoptosis. Studies have shown that when POLQ expression is reduced, DNA damage and apoptosis are increased, and the cellular capacity to end‐join an extrachromosomal substrate with resected ends is reduced.^34^, ^47^, ^48^ The alt‐NHEJ pathway regulated by POLQ maintains cell survival by protecting against catastrophic CIN.^24^ Therefore, although the up‐regulation and down‐regulation of POLQ lead to the similar results, the mechanisms are different. This is the reason why the POLQ inhibition, rather than the promotion should be pursued for SACC treatment.

Accordingly, we propose POLQ depletion to improve SACC sensitivity to DNA damaging agents and prevent SACC progression. An POLQ inhibitor is currently still in research and development; therefore, we used an inhibitor against poly (ADP‐ribose) polymerase 1 (PARP1), a key factor in the alt‐NHEJ pathway. PARP1 inhibitors to suppress the radiotherapy‐ and etoposide‐induced CIN.^42^ PARP1 is an upstream factor in the early alt‐NHEJ pathway, participates in DNA resection, and is highly correlated to DSB repair.^42^ Four PARP inhibitors (PARPi), olaparib, rucaparib, niraparib and talazoparib, have been approved by the US FDA for the treatment of breast cancer, ovarian cancer and peritoneal cancer.^49^ However, there were few study investigating the use of PARPi in SACC research or treatment. Olaparib, the first PARPi approved by FDA that targets PARP1/2, is effective against advanced ovarian cancer and breast cancer.^50^ We found that olaparib suppressed SACC‐83 cell proliferation in a concentration‐dependent manner. The etoposide and olaparib combination suppressed SACC‐83 proliferation, migration and invasion in vitro and inhibited SACC xenograft growth in vivo. These findings suggested that olaparib improved the inhibitory effect of etoposide on SACC.^42^, ^43^ Olaparib inhibits the chromosomal translocation produced by IR or topoisomerase II inhibitor etoposide, suggesting that chromosome translocation is decreased by the suppression on alt‐NHEJ pathway. Therefore, the combined application of olaparib and etoposide has no accumulated effect.

To investigate the mechanism regulating POLQ, we examined the regulators upstream to POLQ. Most DNA polymerases are directly regulated by transcription factors^29^, ^30^; therefore, we speculated that POLQ was regulated by a transcription factor. CCAAT/enhancer binding protein beta (CEBPB) is a bZIP transcription factor in many cellular processes, such as proliferation, differentiation and apoptosis.^51^ Abnormal over‐expression of CEBPB has been reported in many human cancers and is related to cancer progression and poor prognosis. CEPBP is regarded key to inhibit cancer progression and treatment resistance.^52^ Bioinformatics data and the verification experiments suggested that CEBPB directly bond to POLQ promoter. However, we just detected the slight changes of POLQ in the CEBPB‐OE and siCEBPB SACC‐83 cells. We suspect that there are other mechanisms preventing POLQ expression from changing. We plan to identify the relationship between CEBPB and POLQ in future studies.

**FIGURE 10:**
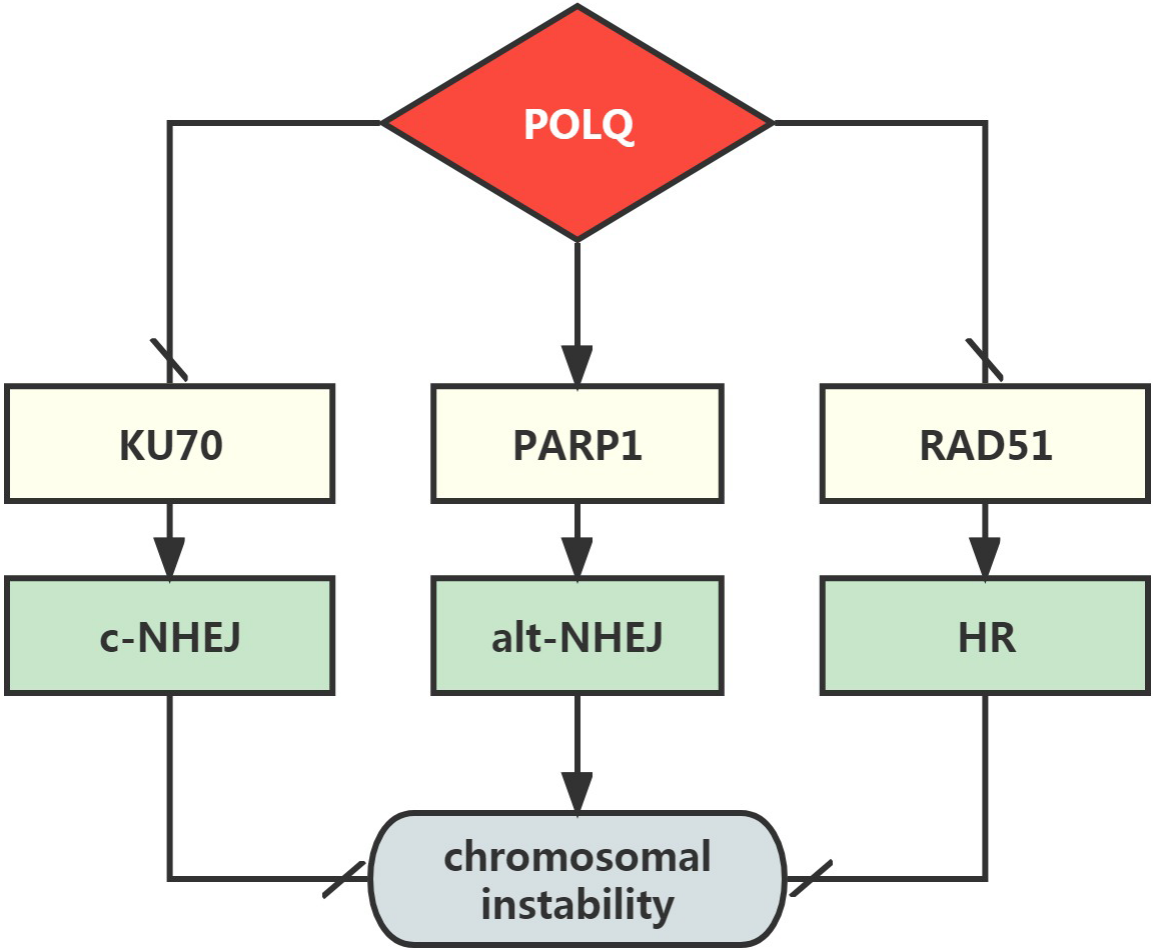
Flow chart of molecular mechanism underlying the effect of POLQ on SACC. POLQ over‐expression inhibited DNA repair factors KU70 and RAD51, and promoted PARP1 in SACC, leading to chromosomal instability and SACC progression

In summary, our results indicate that POLQ inhibits the expressions of DNA repair factors, KU70 and RAD51, but promotes PARP1 expression to regulate DNA damage repair and CIN formation (Figure 10). Therefore, POLQ inhibits the excessive CIN to reduce the risk of cell death and enhance cellular tolerance to DNA damage in SACC. POLQ over‐expression is also a CIN promoting factor by accumulating mutations which promote SACC heterogeneity, evolution and progression. POLQ depletion or olaparib treatment inhibits SACC growth with DNA damaging agent by inducing excessive CIN. Moreover, CEBPB is functionally related with POLQ, which should be pursued in further research.

## AUTHOR CONTRIBUTIONS

**Han Bai:** Data curation (equal); formal analysis (equal); investigation (equal); methodology (equal); software (equal); visualization (equal); writing – original draft (equal); writing – review and editing (equal). **Shilin Xia:** Formal analysis (equal); investigation (equal); methodology (equal); project administration (equal); resources (equal); supervision (equal); validation (equal). **Lei Zhu:** Conceptualization (equal); formal analysis (equal); investigation (equal); methodology (equal); supervision (equal). **Yan Dong:** Conceptualization (equal); data curation (equal); methodology (equal); visualization (equal); writing – review and editing (equal). **Chao Liu:** Methodology (equal); project administration (equal); resources (equal); supervision (equal). **Nan Li:** Methodology (equal); project administration (equal); resources (equal); supervision (equal). **Han Liu:** Conceptualization (equal); data curation (equal); funding acquisition (equal); investigation (equal); methodology (equal); project administration (equal); supervision (equal); writing – review and editing (equal). **Jing Xiao:** Conceptualization (equal); formal analysis (equal); funding acquisition (equal); methodology (equal); project administration (equal); resources (equal); supervision (equal); writing – review and editing (equal).

## CONFLICT OF INTEREST

None of authors have conflicts of interest to declare.

## Supporting information


Appendix S1

## Data Availability

The datasets used and/or analysed during the current study are available from the corresponding author on reasonable request.
