# Supplementary material for: Altered polymerase theta expression promotes chromosomal instability in salivary adenoid cystic carcinoma
Source: J Cell Mol Med. 2022 Jun 21;26(14):3931–49. doi: 10.1111/jcmm.17429 (PMC9279586; doi:10.1111/jcmm.17429)
Supplement: Supplementary file 1 — Appendix S1 [file JCMM-26-3931-s001.doc]

**Supplementary Table 1.** Primer sequences for qRT-PCR

| **Target** | **Primer** | **Sequence** |
| --- | --- | --- |
| *GAPDH* | Forward | 5'-GCACCGTCAAGGCTGAGAAC-3' |
|  | Reverse | 5'-TGGTGAAGACGCCAGTGGA-3' |
| *POLQ* | Forward | 5'-CAGTGAGCATTCTGGGCGATA-3' |
|  | Reverse | 5'-AGCAAGGCCAAGCAGTACTGA-3' |
| *MLH1* | Forward | 5'-TCCAGCAACCCCAGAAAGA-3' |
|  | Reverse | 5'-GATTCACACAGCCCACGAAG-3' |
| *CENP-A* | Forward | 5'-TGCACCCAGTGTTTCTGTCAGTC-3' |
|  | Reverse | 5'-ATTGGATCTAGTCATGGCTCTGGA-3' |
| *HJURP* | Forward | 5'-CATGCAGCGGCTGATAGAGA-3' |
|  | Reverse | 5'-TAGGTCAGCGTGGCCATTTG-3' |
| *CEBPB* | Forward | 5'-TTTGTCCAAACCAACCGCAC-3' |
|  | Reverse | 5'-GCATCAACTTCGAAACCGGC-3' |

**Supplementary Table 2. Primary antibodies used for western blot analysis**

| **Target** | **Company** | **Catalog number** | **Dilution** |
| --- | --- | --- | --- |
| POLQ | Invitrogen | PA5-69577 | 1:500 |
| GAPDH | Beyotime | AG019 | 1:1000 |
| VINCULIN | Proteintech | 66305-1-Ig | 1:1000 |
| γH2AX | Abcam | ab2893 | 1:1000 |
| KU70 | Proteintech | 10723-1-AP | 1:1000 |
| RAD51 | Abcam | ab213 | 1:250 |
| PARP1 | Abcam | ab32138 | 1:1000 |
| MLH1 | Proteintech | 11697-1-AP | 1:1000 |
| CENP-A | ABclone | A15995 | 1:500 |

**Supplementary Table 3.** Primer sequences for PCR-SSCP

| **Target** | **Primer** | **Sequence** |
| --- | --- | --- |
| *BAT-25* | Forward | 5'-TCGCCTCCAAGAATGTAAGT-3' |
|  | Reverse | 5'-TCTGCATTTTAACTATGGCTC-3' |
| *BAT-26* | Forward | 5'-TGACTACTTTTGACTTCAGCC-3' |
|  | Reverse | 5'-AACCATTCAACATTTTTAACCC-3' |
| *D2S123* | Forward | 5'-AAACAGGATGCCTGCCTTTA-3' |
|  | Reverse | 5'-GGACTTTCCACCTATGGGAC-3' |
| *D17S250* | Forward | 5'-GGAAGAATCAAATAGACAA-3' |
|  | Reverse | 5'-GCTGGCCATATATATATTTAAACC-3' |
| *D5S346* | Forward | 5'-ACTCACTCTAGTGATAAATCG-3' |
|  | Reverse | 5'-AGCAGATAAGACAGTATTACTAGTT-3' |


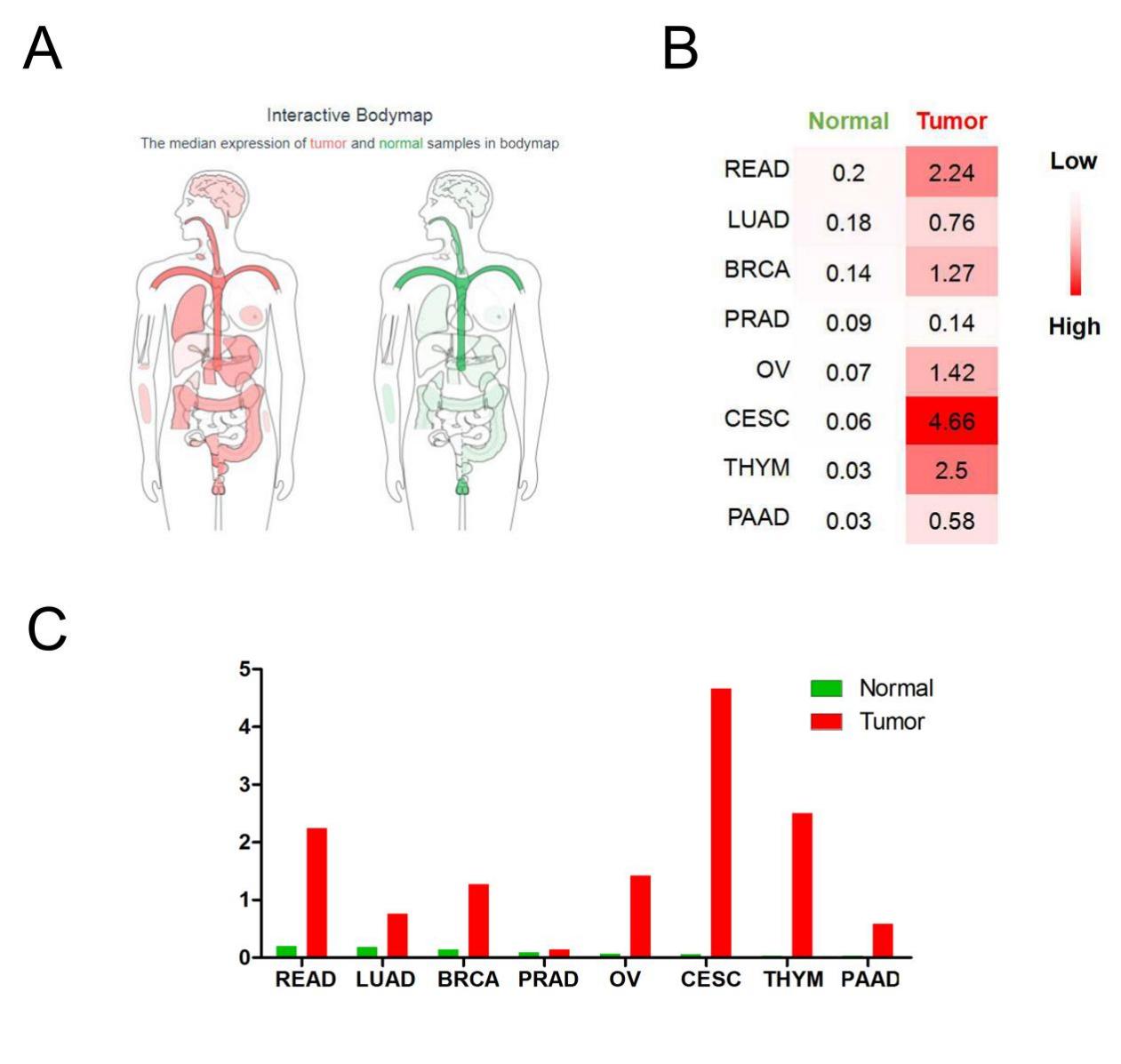


**Supplementary Figure 1.** The expression of POLQ in tumor and corresponding normal tissues. A Illustration of the median expression of POLQ in tumor and normal samples. B Heatmap of the median expression of POLQ in tumor and normal samples. C Histogram of the median expression of POLQ in tumor and normal samples. (READ: rectum adenocarcinoma; LUAD: lung adenocarcinoma; BRCA: breast invasive carcinoma; PRAD: prostate adenocarcinoma; OV: ovarian serous cystadenocarcinoma; CESC: cervical squamous cell carcinoma and endocervical adenocarcinoma; THYM: thymoma; PAAD: pancreatic adenocarcinoma).


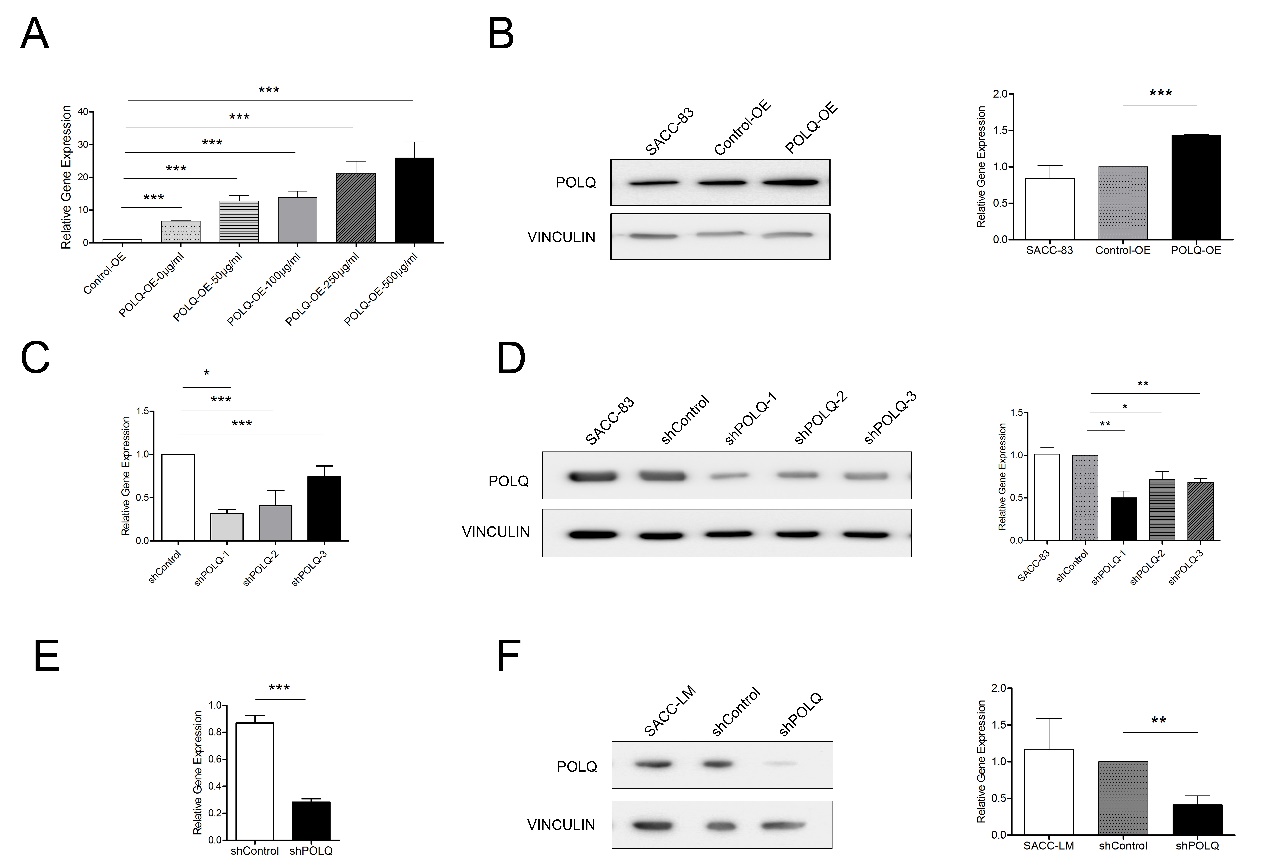


**Supplementary Figure 2.** Construction of SACC cells with the altered POLQ expression. **A** qRT-PCR for POLQ mRNA in the indicated SACC-83 cells selected by different antibiotic concentrations. **B** Western blots and quantification of POLQ in the indicated cells treated with 500 µg/ml hygromycin B for 48 h. **C** qRT-PCR for POLQ mRNA in the indicated SACC-83 cells. **D** Western blots and quantification of POLQ in the indicated cells. **E** qRT-PCR for POLQ mRNA in the indicated (SACC-LM) cells. **F** Western blots and quantification of POLQ in the indicated cells. Error bars in graphs reflect SEM; **p* < 0.05, ***p* < 0.01,****p*< 0.001, determined by unpaired, two-tailed t-test.


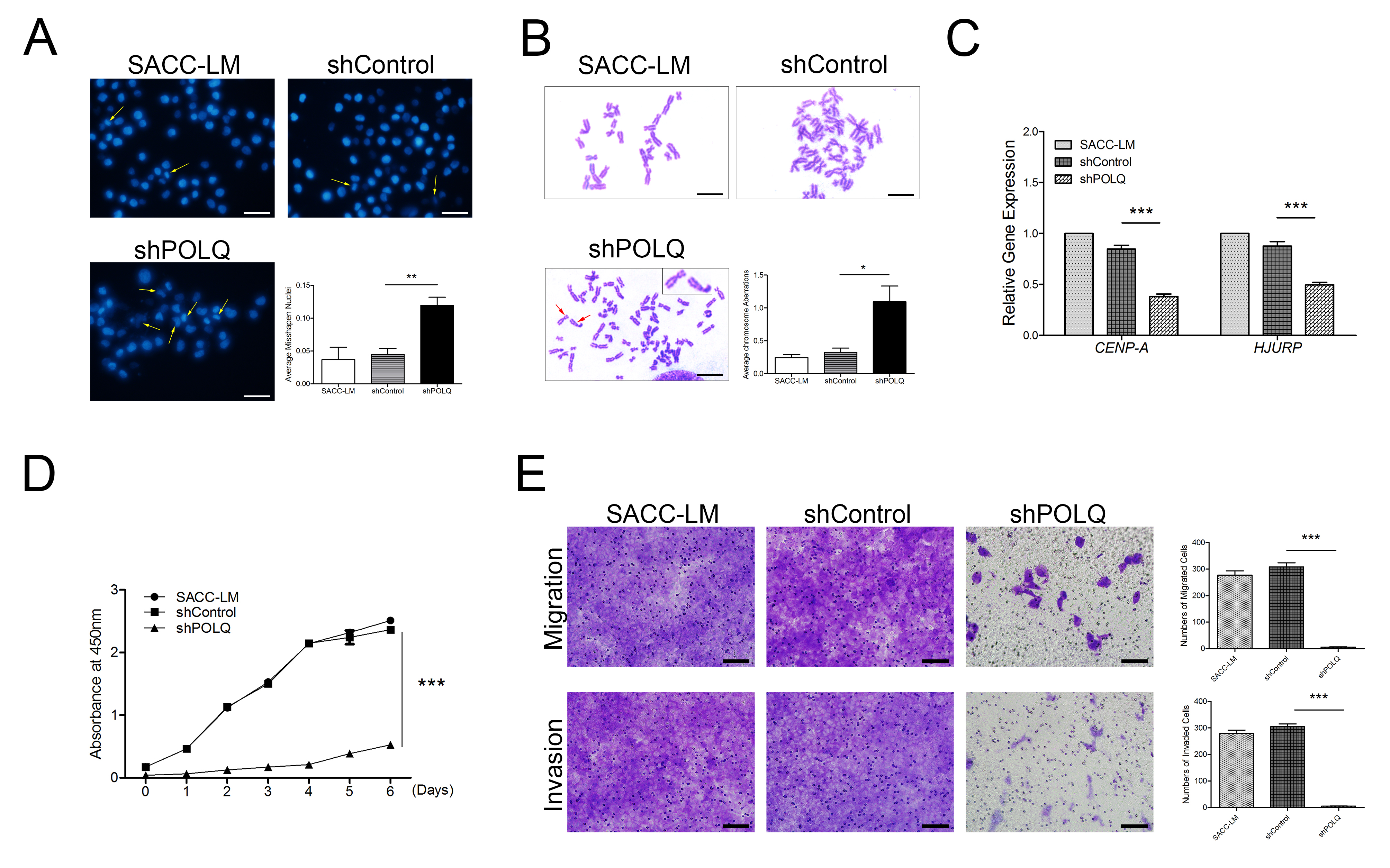


**Supplementary Figure 3.** Effects of altered POLQ expression on GIN and

proliferation and migration/invasion in SACC-LMs. **A** Representative images of cells with DAPI staining of nuclei, and quantification of nucleus deformation (indicated by yellow arrows in images) in the indicated cells. Scale bars, 50 μm. **B** Representative images of chromosomal aberrations (indicated by red arrows in images) and quantification of chromosomal aberrations in the indicated cells. Scale bars, 10 μm. **C** qRT-PCR for CENP-A and HJURP mRNAs in the indicated cells. **D** Cell proliferation of the indicated cells was determined by CCK-8 assay. **E** Transwell migration (top panel) and invasion assays (bottom panel) with the indicated cells. Scale bars, 100 μm. Error bars in charts reflect SEM; **p* < 0.05, ***p* < 0.01, ****p* < 0.001, determined by unpaired, two-tailed t-test.


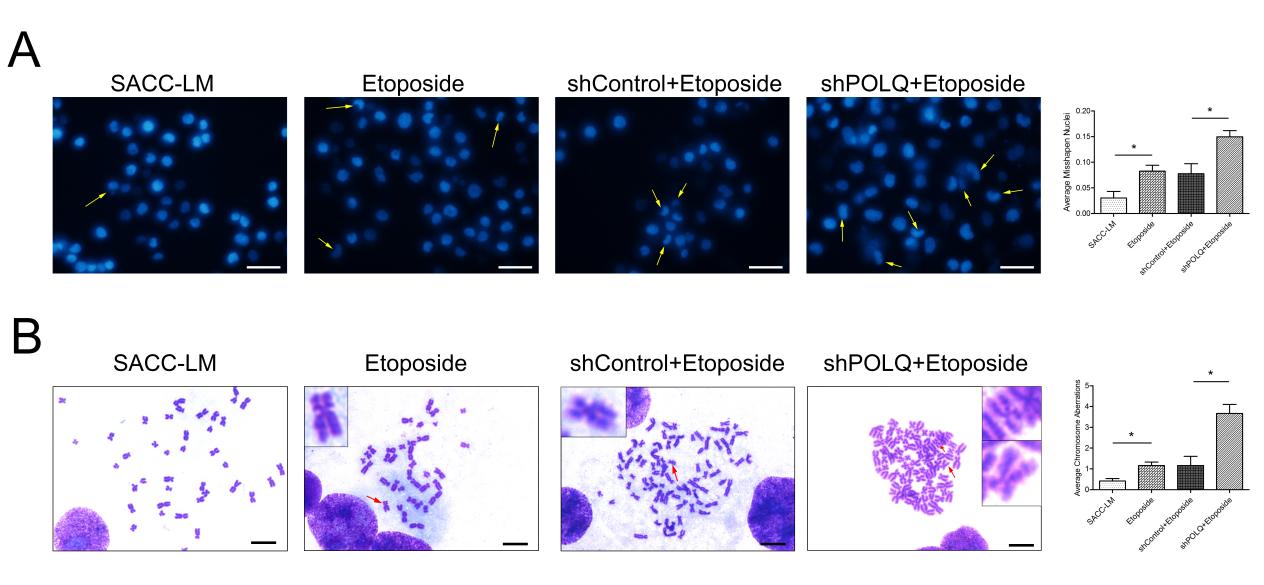


**Supplementary Figure 4.** Effects of altered POLQ expression on chromosomal stability under etoposide-induced DNA damage in SACC-LM cells. **A**

Representative images (left panel) of cells with DAPI staining of nuclei and quantification (right panel) of nucleus deformation (indicated by yellow arrows in images) in the indicated cells. Scale bars, 50 μm. **B** Representative images (left panel) of chromosomal aberrations (indicated by red arrows) and quantification (right panel) of chromosomal aberrations in the indicated cells. Scale bars, 10 μm. Error bars in graph reflect SEM; **p* < 0.05, determined by unpaired, two-tailed t-test.
